# Pan-Cancer Analysis of the Solute Carrier Family 39 Genes in Relation to Oncogenic, Immune Infiltrating, and Therapeutic Targets

**DOI:** 10.3389/fgene.2021.757582

**Published:** 2021-12-02

**Authors:** Yi-Yuan Qu, Rong-Yan Guo, Meng-Ling Luo, Quan Zhou

**Affiliations:** ^1^ Department of Gynecology and Obstetrics, the People’s Hospital of China Three Gorges University/The First People’s Hospital of Yichang, Yichang, China; ^2^ Emergency Services Department, HanYang Hospital Affiliated of Wuhan University of Science and Technology, Wuhan, China

**Keywords:** *SLC39* family genes, biomarker, pan-cancer, prognosis, immune infiltration, drug sensitivity

## Abstract

**Background:** Emerging pieces of evidence demonstrated that the solute carrier family 39 (SLC39A) members are critical for the oncogenic and immune infiltrating targets in multiple types of tumors. However, the precise relationship between the *SLC39A* family genes and clinical prognosis as well as the pan-cancer tumor cell infiltration has not been fully elucidated.

**Methods:** In this study, the pan-cancer expression profile, genetic mutation, prognostic effect, functional enrichment, immune infiltrating, and potential therapeutic targets of the SLC39A family members were investigated by analyzing multiple public databases such as the Oncomine, TIMER, GEPIA, cBioPortal, KM-plotter, PrognoScan, GeneMANIA, STRING, DAVID, TIMER 2.0, and CellMiner databases.

**Results:** The expression levels of most *SLC39* family genes in the tumor tissues were found to be significantly upregulated compared to the normal group. In mutation analysis, the mutation frequencies of *SLC39A4* and *SLC39A1* were found to be higher among all the members (6 and 4%, respectively). Moreover, the overall mutation frequency of the *SLC39A* family genes ranged from 0.8 to 6% pan-cancer. Also, the function of the *SLC39A* highly related genes was found to be enriched in functions such as zinc II ion transport across the membrane, steroid hormone biosynthesis, and chemical carcinogenesis. In immune infiltration analysis, the expression level of the SLC39A family genes was found to be notably related to the immune infiltration levels of six types of immune cells in specific types of tumors. In addition, the *SLC39A* family genes were significantly related to the sensitivity or resistance of 63 antitumor drugs in a variety of tumor cell lines.

**Conclusion:** These results indicate that the *SLC39* family genes are significant for determining cancer progression, immune infiltration, and drug sensitivity in multiple cancers. This study, therefore, provides novel insights into the pan-cancer potential targets of the *SLC39* family genes.

## Introduction

Cancer has gradually emerged as the leading threat to public health worldwide, as estimated by GLOBOCAN 2020, stating 19.3 million newly confirmed cancer cases and nearly 10 million cancer-related deaths (Ferlay et al., 2021; Sung et al., 2021). Indeed, efforts for cancer prevention, screening, diagnosis, and comprehensive treatment have met with tremendous success in various tumors. However, studies on the clinical outcome of most cancers need further improvisation (Chen et al., 2021). The current promising targeted therapy particularly confirms that exploring the mechanism of pan-cancer initiation, maintenance, and development will unfurl new avenues for fighting various malignant tumors (Loomans-Kropp and Umar, 2019). Therefore, identification of the hub tumor-related genes is very urgent and necessary to develop new diagnostic and prognostic biomarkers and therapeutic targets. Presently, massive high-throughput data and multiple available big data online public databases are greatly helpful for finding the tumorigenic genes and conducting pan-cancer studies in multi-omics (Loomans-Kropp and Umar, 2019; Xiao et al., 2021).

The *SLC39A* family genes encode a family of proteins belonging to the Zrt- and Irt-like protein (ZIP) transport proteins, having 14 family members (SLC39A1-14). It controls the transportation and influx of zinc, with important roles in multiple signaling pathways and physiological processes, like gene transcription, endocrine regulation, cell growth, cell differentiation, and the immune response process (Kimura and Kambe, 2016; Baltaci and Yuce, 2018). Emerging pieces of evidence indicate that the mutation or functional change in the *SLC39A* family genes leads to the development and progression of multiple malignancies, such as colorectal cancer, breast cancer, esophageal cancer, hepatocellular carcinoma, pancreas cancer, gastric cancer, prostate cancer, and lung cancer (Hoang et al., 2016; To et al., 2020; Prasad, 2012). Besides, recent multi-omics studies have confirmed certain SLC39A family genes to have differential expression and prognostic value in breast, gastric, and lung cancers, acting as potentially promising clinical markers for these cancers (Liu et al., 2020; Zhou et al., 2021; Ding et al., 2019). Some basic studies have demonstrated the targeted regulation of the *SLC39A* family genes to be capable of changing the biological characteristics of some tumor cells. For example, Jin et al. found that knockdown of SLC39A5 expression significantly inhibits the invasion, proliferation, and migration of esophageal tumor cells (Jin et al., 2015). In addition, Zhu et al. demonstrated the knockdown of SLC39A11 to attenuate the cellular proliferation of the pancreatic cancer Capan-1 with decreased activation of the ERK1/2 pathway (Zhu et al., 2021). Fan et al. have found *SLC39A4* gene knockout to inhibit the malignant behavior of the ovarian tumor cells both *in vitro* and *in vivo* (Fan et al., 2017). More importantly, the growing studies have shown that SLC transporters not only directly bring the anticancer drugs into cancer cells but also serve as a medium for the uptake of essential nutrients for the growth and survival of the tumor, thereby regulating the sensitivity and resistance of the chemotherapeutic drugs (Li and Shu, 2014). Nevertheless, the underlying mechanism and biological functions of the *SLC39A* family genes in the tumor progression and as the potential therapeutic target have not been fully elucidated.

This study systematically performed an in-depth analysis on the expression of the *SLC39A* family genes and their impact on the prognosis, to explore the relationship between the *SLC39A* family genes and pan-cancer immune cell infiltration. In addition to utilizing the multi-omics and large sample data analysis, the genetic mutation, function enrichment, and drug sensitivity of the *SLC39A* family genes were investigated across different cancer types. These analyses could provide a new direction for a promising biomarker and potential targeted therapy for treating cancer.

## Materials and Methods

### Expression Profiles Analysis

Three online databases (Oncomine, TIMER, and GEPIA) were applied to investigate the differential expression profiles of the SLC39A family genes between the normal and the tumor tissues in various cancer types. The website of the Oncomine online platform is www.oncomine.org (Rhodes et al., 2007), the website of the TIMER online platform is https://cistrome.shinyapps.io/timer/ (Li et al., 2017), and the website of the GEPIA online platform is http://gepia.cancer-pku.cn/ (Tang et al., 2017). Among them, the *p*-value was set to 0.01; fold change was set to 1.5; the gene level was set to all, and data type was set to mRNA in the Oncomine database, and the relevant parameters of the TIMER and GEPIA databases were set by default.

### Mutation Profiles Analysis

The cBioPortal (http://www.cbioportal.org) (Gao et al., 2013) was exploited for detecting the mutation landscape (amplification, deep deletion, and missense mutations) and general mutation count of the *SLC39A* family genes in 33 types of tumors from the TCGA database. In addition, the impact of gene mutations in the *SLC39A* family on the clinical outcomes was surveyed using the cBioPortal database.

### Survival Analysis

The relationship between the expression of the *SLC39A* family gene and the overall survival (OS) and progression-free survival (PFS) in the pan-cancer patients was investigated using the pan-cancer module of the KM-plotter database (http://www.kmplot.com/) (Nagy et al., 2021). In addition, the PrognoScan database (http://dna00.bio.kyutech.ac.jp/PrognoScan/index.html) (Mizuno et al., 2009) was further utilized to confirm the relationship between the expression of the SLC39A family genes and clinical outcome in the different cohorts. Multiple types of survival parameters, including OS, PFS, relapse-free survival (RFS), disease-free survival (DFS), distant recurrence–free survival (DRFS), distant metastasis–free survival (DMFS), and disease-specific survival (DSS) were represented in the current analysis. The hazard ratio (HR), log-rank *p-*value, and 95% confidence interval were directly displayed on the online platform, and the *p-*value cut-off value was set to 0.05.

### Enrichment Analysis

To seek out the highly related genes of SLC39A family genes, the GeneMANIA database (http://www.genemania.org) (Warde-Farley et al., 2010) and the STRING database (https://string-db.org) (Szklarczyk et al., 2019) were exploited. Then, the DAVID database (Database for Annotation, Visualization, and Integrated Discovery, https://david.ncifcrf.gov) (Huang et al., 2009) was used to conduct the GO (gene ontology) annotation and KEGG (Kyoto Encyclopedia of Genes and Genomes) enrichment analysis of the *SLC39A* family highly related genes.

### Immune Infiltration Analysis

The TIMER 2.0 (http://cistrome.shinyapps.io/timer) (Li et al., 2020) was used to evaluate the relationship between the *SLC39A* family gene expression levels and the infiltration of six common immune cells, including the B cells, CD4^+^ T cells, CD8^+^ T cells, Treg T cells, macrophages, and neutrophils.

### Drug Sensitivity Analysis

The CellMiner database (https://discover.nci.nih.gov/cellminer/) (Shankavaram et al., 2009) was exploited to evaluate the relationship between the *SLC39A* family gene expression levels and the compound sensitivity or resistance through the NCI-60 analyses tools. Data processing and Pearson correlation analysis visualization used the limma and ggplot2 package, and the scatter plot showed significant correlations sorted by *p*-value from small to large, and the *p*-value cut-off value was set to 0.05.

## Results

### The Expression Profiles of the SLC39A Family Genes in Pan-Cancer

**FIGURE 1:**
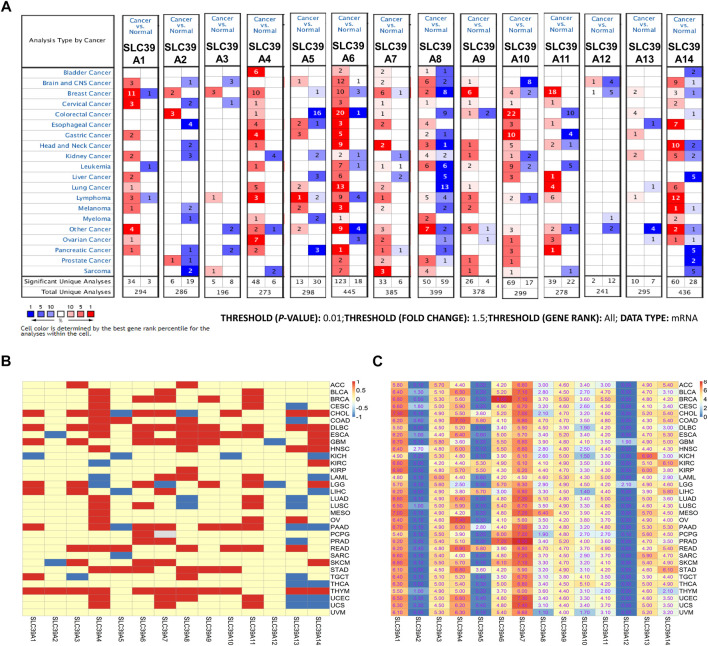
mRNA expression profiles of the *SLC39A* family genes in pan-cancer. **(A)** Transcriptome expression profile of SLC39A family members in pan-cancer was explored in the Oncomine database. In the graph, red represents statistically significant mRNA overexpression of *SLC39A* family gene mRNA between the tumor and the corresponding normal tissue, blue represents down-expression, and the number represents the number of data sets. *p* value is set to 0.01; fold change is set to 1.5; gene level is set to all; and data type is set to mRNA in the Oncomine database. **(B)** Transcriptome expression profiles of *SLC39A* family genes in pan-cancer were explored in the GEPIA database. The red boxes represent higher *SLC39A* family gene expression in tumor tissues, while the green boxes represent the lower *SLC39A* family gene expression in tumor tissues. The inspection standard is set to *p*-value < 0.05. **(C)** Expression level of *SLC39A* family genes in 33 tumors and their normal controls in the match TCGA normal and GTEx data using GEPIA. The data in the figure represents average mRNA expression of *SLC39A* family genes in different tumors. The colors from blue to red represent the range of values in the figure.

**FIGURE 2:**
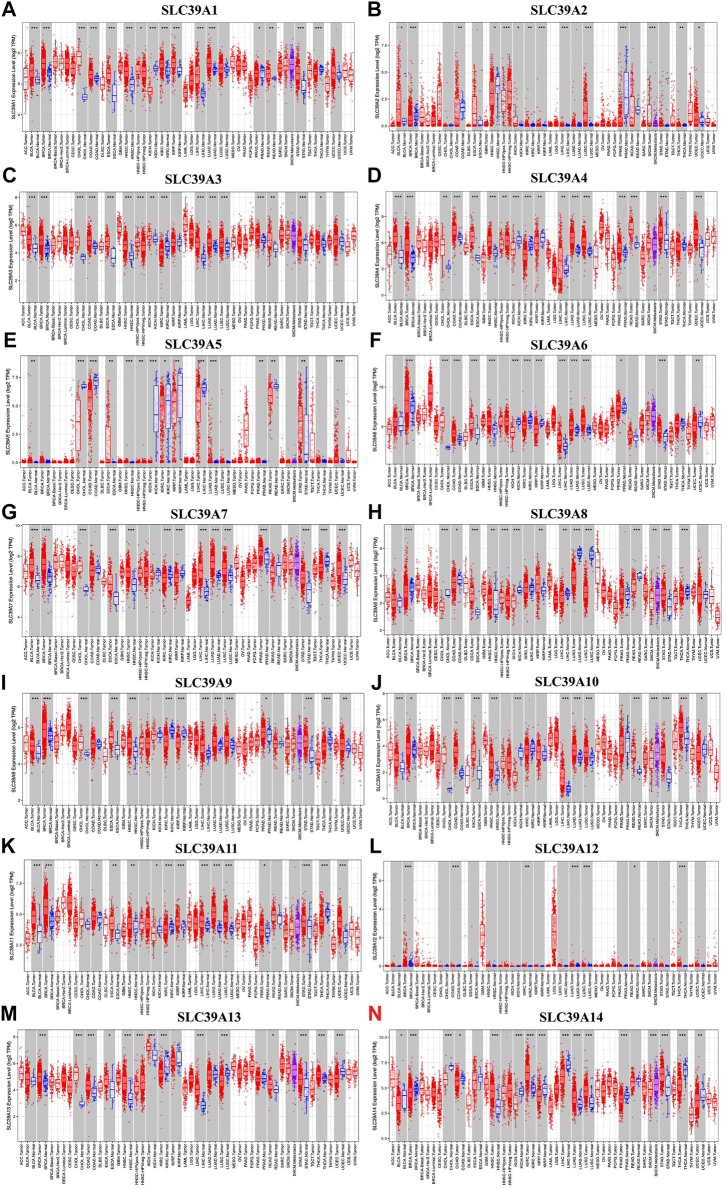
Differential expression of the *SLC39A* family genes in pan-cancer and corresponding normal tissues. **(A)** Transcriptome expression of *SLC39A1* was explored in the TIMER database. **(B)** Transcriptome expression of *SLC39A2* was explored in the TIMER database. **(C)** Transcriptome expression of *SLC39A3* was explored in the TIMER database. **(D)** Transcriptome expression of *SLC39A4* was explored in the TIMER database. **(E)** Transcriptome expression of *SLC39A5* was explored in the TIMER database. **(F)** Transcriptome expression of *SLC39A6* was explored in the TIMER database. **(G)** Transcriptome expression of *SLC39A7* was explored in the TIMER database. **(H)** Expression of *SLC39A8* was explored in the TIMER database. **(I)** Transcriptome expression of *SLC39A 9* was explored in the TIMER database. **(J)** Transcriptome expression of *SLC39A10* was explored in the TIMER database. **(K)** Transcriptome expression of *SLC39A11* was explored in the TIMER database. **(L)** Transcriptome expression of *SLC39A12* was explored in the TIMER database. **(M)** Transcriptome expression of *SLC39A13* was explored in the TIMER database. **(N)** Transcriptome expression of *SLC39A14* was explored in the TIMER database. (*p < 0.05, **p < 0.01, ***p < 0.001. The red box and the green box represent tumor tissue and normal control tissue, respectively. The middle line of the box represents the median and the lower and upper bounds represent the 25th and 75th percentiles, respectively.)

Subsequently, the expression profiles of the *SLC39A* family genes were explored in various cancer types. The Oncomine, GEPIA, and TIMER databases were exploited to examine and verify the expression levels of the *SLC39A* family genes in the tumor tissues and the corresponding non-tumor tissues. The Oncomine database reported an increase in the mRNA expression level of other *SLC39* family genes in the tumor tissues compared to the normal control group, except for *SLC39A8* (Figure 1A). The median expression of the *SLC39A* family genes in the tumor tissues of all types of tumors was further compared, revealing that most of the *SLC39A* family genes show relatively high expression in the specific tumor types, such as lymphoid neoplasm diffuse large B-cell lymphoma (DLBC), esophageal carcinoma (ESCA), glioblastoma multiforme (GBM), head and neck squamous cell carcinoma (HNSC), rectum adenocarcinoma (READ), and thymoma (THYM) (Figure 1B). In addition, the expression level of the *SLC39A* family genes was detected in 33 tumors, and their normal controls match TCGA normal and GTEx data using GEPIA. Similar to the results of the other studies, the *SLC39A* family genes have notably increased the expression in most tumors compared to the normal controls (Figure 1C and Supplementary Table S1). As shown in Figure 2, the TIMER2.0 database results demonstrated that the transcriptional expression levels of the *SLC39A* family genes are inconsistent between the tumor tissues and corresponding normal tissues, and most SLC39A families were over-regulated in the tumor tissues, extremely so in the bladder urothelial carcinoma (BLCA), breast invasive carcinoma (BRCA), cholangiocarcinoma (CHOL), colon adenocarcinoma (COAD), HNSC, kidney renal papillary cell carcinoma (KIRP), lung adenocarcinoma (LUAD), stomach adenocarcinoma (STAD), and thyroid carcinoma (THCA) tumor types.

### The Genetic Mutation of the SLC39A Family Genes in Pan-Cancer

**FIGURE 3:**
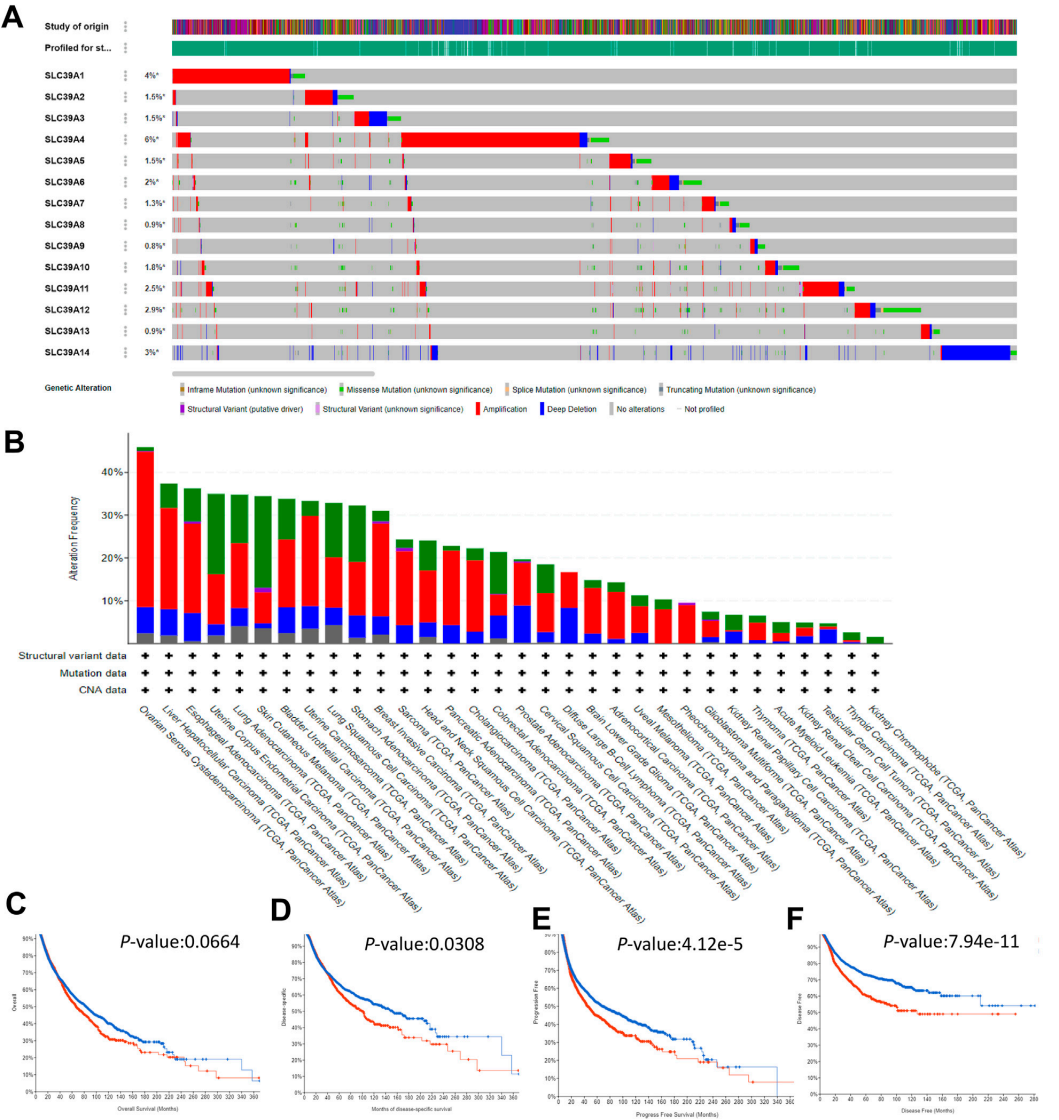
Mutation landscape of the *SLC39A* family genes in pan-cancer derived from the cBioPortal platform. **(A)** OncoPrint summary of alteration on *SLC39A* family genes in 10,967 numbers of samples in 32 studies of the pan-cancer atlas. The mutation frequency was shown as green for mutations, red for fusions, blue for sions, and black for multiple mutations. **(B)** Rectangular graph of the general mutation counts of *SLC39A* family genes in the pan-cancer atlas. The *X*- and *Y*-axis represent the mutation frequency of *SLC39A* family genes and cancer type, respectively. It was shown as green for missense mutations, violet for fusions, deep blue for truncating, and blue for no mutations. **(C)** Kaplan–Meier chart of OS of pan-cancer with and without *SLC39A* family gene mutation. **(D)** Kaplan–Meier chart of DDS of pan-cancer with and without *SLC39A* family gene mutation. **(E)** Kaplan–Meier chart of PFS of pan-cancer with and without *SLC39A* family gene mutation. **(F)** Kaplan–Meier chart of DFS of pan-cancer with and without *SLC39A* family gene mutation. The red line and the blue line represent high and low expression, respectively. The Cox *p*-value cut-off value was set to 0.05.

The cBioPortal and TCGA database was employed to probe the mutation status of the *SLC39A* family genes in 10,967 samples in 32 studies of the pan-cancer atlas. Results showed that the mutation frequencies of *SLC39A4* and *SLC39A1* were higher than those of all the other members, 6 and 4%, respectively, and the overall mutation frequency of the *SLC39A* family genes ranged from 0.8 to 6% (Figure 3A). As shown in Figure 3B, the mutation frequency of the *SLC39A* family genes in ovarian serous cystadenocarcinoma (OV), liver hepatocellular carcinoma (LIHC), ESCA, uterine corpus endometrial carcinoma (UCEC), LUAD, skin cutaneous melanoma (SKCM), BLCA, uterine carcinosarcoma (UCS), lung squamous cell carcinoma (LUSC), STAD, and BRCA was relatively higher by more than 30%, and the other types of tumors all exhibited a very low alteration in mutation (<30%). In addition, the Kaplan–Meier plotter results demonstrated that the combined mutation of the *SLC39A* family genes has no significant effect on OS (*p*-values, 0.0664) (Figure 3C). However, there are statistical differences in the DSS, PFS, and DFS between the mutation group and the non-mutation group of the *SLC39A* family genes (*p*-values, 0.0308, 4.11e-5, and 7.94e-11, respectively) (Figures 3D–F).

### The Prognostic Value of SLC39A Family Genes in Pan-Cancer

**FIGURE 4:**
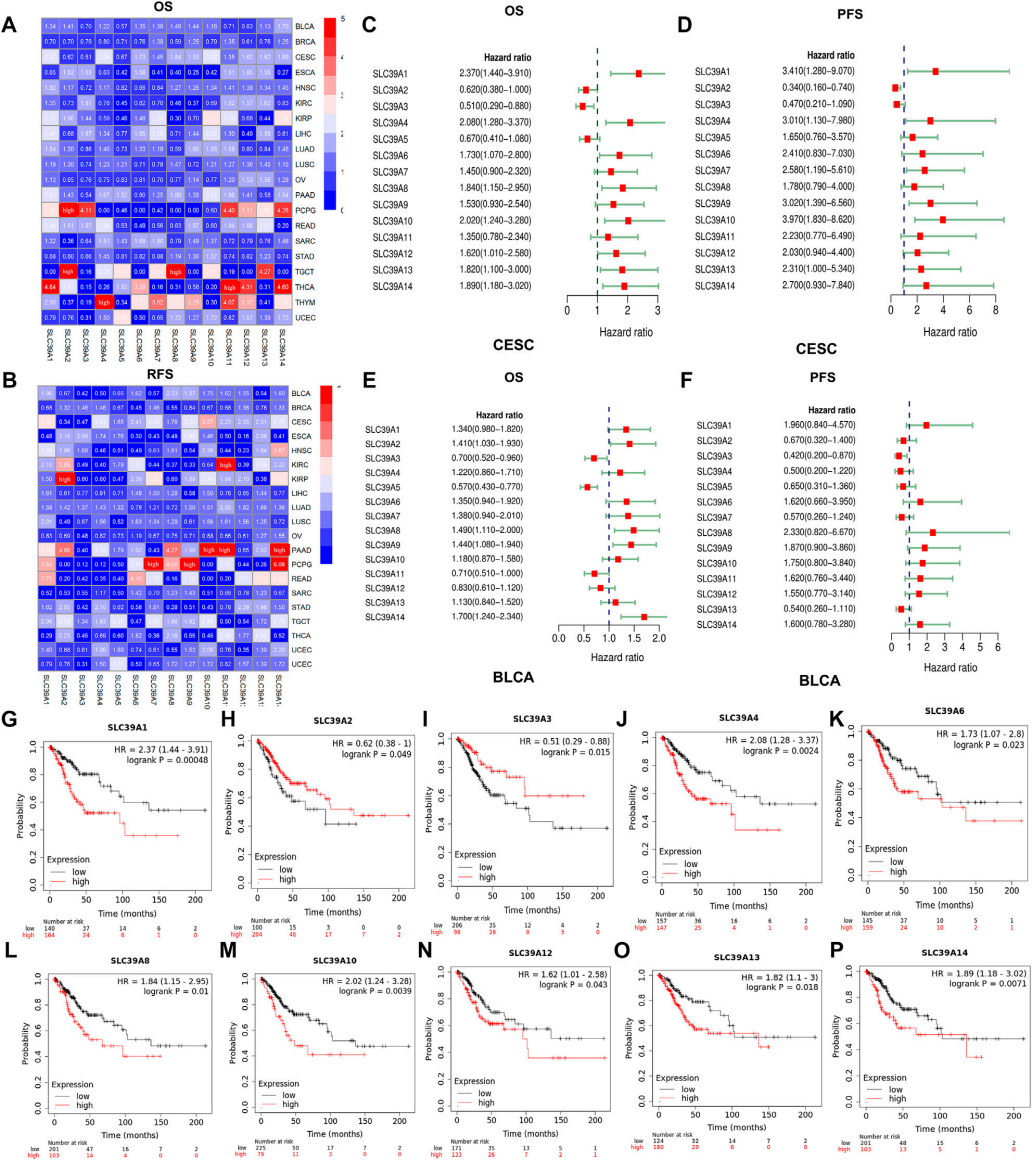
Survival analysis of the prognostic value of the *SLC39A* family genes in pan-cancer derived from the KM-plotter dataset. **(A)** Heat map shows the hazard ratio (HR) value of the OS in pan-cancer calculated using the KM-plotter database. **(B)** Heat map shows the hazard ratio (HR) value of the RFS in pan-cancer calculated using the KM-plotter database. (The colors from blue to red represent the range of HR values in the figure. HRs over 5 were replaced with “high.”) **(C)** Forest plot quantitatively synthesizes the HR and 95% confidence interval of the OS of the *SLC39A* gene family in CESC. **(D)** Forest plot quantitatively synthesizes the HR and 95% confidence interval of the PFS of the *SLC39A* gene family in CESC. **(E)** Forest plot quantitatively synthesizes the HR and 95% confidence interval of the OS of the *SLC39A* gene family in BLCA. **(F)** Forest plot quantitatively synthesizes the HR and 95% confidence interval of the PFS of the *SLC39A* gene family in BLCA. **(G)** Survival curve of *SLC39A1* on the OS in CESC. **(H)** Survival curve of *SLC39A2* on the OS in CESC. **(I)** Survival curve of *SLC39A3* on the OS in CESC. **(J)** Survival curve of *SLC39A4* on the OS in CESC. **(K)** Survival curve of *SLC39A6* on the OS in CESC. **(L)** Survival curve of *SLC39A8* on the OS in CESC. **(M)** Survival curve of *SLC39A10* on the OS in CESC. **(N)** Survival curve of *SLC39A12* on the OS in CESC. **(O)** Survival curve of *SLC39A13* on the OS in CESC. **(P)** Survival curve of *SLC39A14* on the OS in CESC. The red line and the blue line represent high and low expression, respectively. The Cox *p*-value cut-off value was set to 0.05.

The association between the mRNA expression of the *SLC39A* family genes and the clinical outcomes in pan-cancer patients were analyzed using the KM-plotter and PrognoScan databases. As shown in Figure 4, the KM-plotter database revealed that the expression of the *SLC39A* family genes was significantly related to the OS and RFS in some tumor types. Among them, the high expression of most of the *SLC39A* family genes presents the risk factor for the OS of BLCA, CESC, HNSC, LIHC, LUAD, LUSC, and PAAD, as well as for the protection factors for OS of BRCA, ESCA, OV, PAAD, STAD, TGCT, and THCA. Similarly, for RFS, the high expression of most *SLC39A* family genes was significantly related to the inferior survival of BLCA, CESC, KIRP, LUAD, LUSC, PAAD, and TGCT, as well as the better prognosis of BRCA, OV, PCPG, and STAD (Figures 4A,B and Supplementary Tables S2, S3). As shown in Figures 4C–R, the increase in the expression of SLC39A1, SLC39A 3, SLC39A 5, SLC39A 8, SLC39A 10, SLC39A 13, and SLC39A 14 was associated with poor OS, and the upregulation of SLC39A1, SLC39A 4, SLC39A 7, SLC39A 9, and SLC39A 10 was found to lead to poor RFS in the patients with CESC.

**FIGURE 5:**
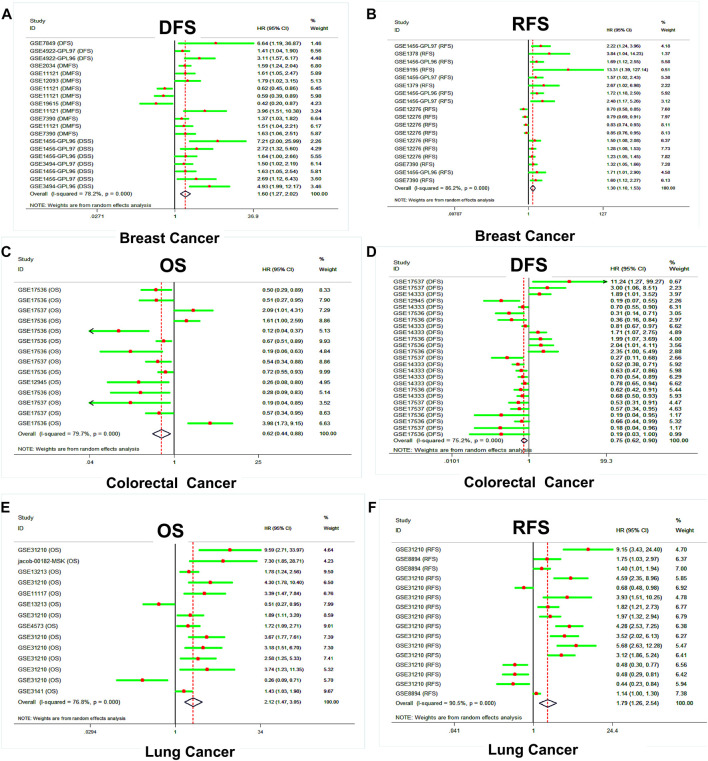
Survival analysis of the prognostic value of the *SLC39A* family genes in pan-cancer derived from the PrognoScan dataset. **(A)** Forest plot of DDS shows the effect of *SLC39A* family gene expression on the clinical prognosis of breast cancer. **(B)** Forest plot of RFS shows the effect of *SLC39A* family gene expression on the clinical prognosis of breast cancer. **(C)** Forest plot of OS shows the effect of *SLC39A* family gene expression on the clinical prognosis of colorectal cancer. **(D)** Forest plot of DFS shows the effect of *SLC39A* family gene expression on the clinical prognosis of colorectal cancer. **(E)** Forest plot of OS shows the effect of *SLC39A* family gene expression on the clinical prognosis of lung cancer. **(F)** Forest plot of DFS shows the effect of *SLC39A* family gene expression on the clinical prognosis of lung cancer.

Then, the PrognoScan platform was used to further assess and verify the prognostic value of the *SLC39A* family genes in pan-cancer, based on public datasets. The results of PrognoScan indicated that the expression level of the *SLC39A* family genes was significantly related to the clinical survival of 12 types of tumors, such as colorectal cancer, breast cancer, bladder cancer, lung cancer, ovarian cancer, blood cancer, brain cancer, skin cancer, eye cancer, soft tissue cancer, prostate cancer, and head and neck cancer (Supplementary Table S4). Interestingly, most of the studies and data sets were previously focused on breast, colorectal, lung, and ovarian cancer. The results of the quantitative synthesis of related studies showed that higher expression of the *SLC39A* family genes indicated a worse survival prognosis for RFS ([HR] = 1.30, 95% confidence interval [CI] = 1.10 to 1.53) and DSS (HR = 1.60, 95% CI = 1.27 to 2.02) in breast cancer (Figures 5A,B). However, the higher expression of the *SLC39A* family genes was associated with a better prognosis for OS (HR = 0.62, 95% CI = 0.44 to 0.88) and DFS (HR = 0.75, 95% CI = 0.62 to 0.90) in colorectal cancer (Figures 5C,D). In addition, the upregulation of the *SLC39A* family gene expression was significantly associated with poor OS (HR = 2.12, 95% CI = 1.45 to 3.05) and RFS (HR = 1.79, 95% CI = 1.26 to 2.54) in lung cancer.

### The Function Enrichment of the SLC39A Family Genes in Pan-Cancer

**FIGURE 6:**
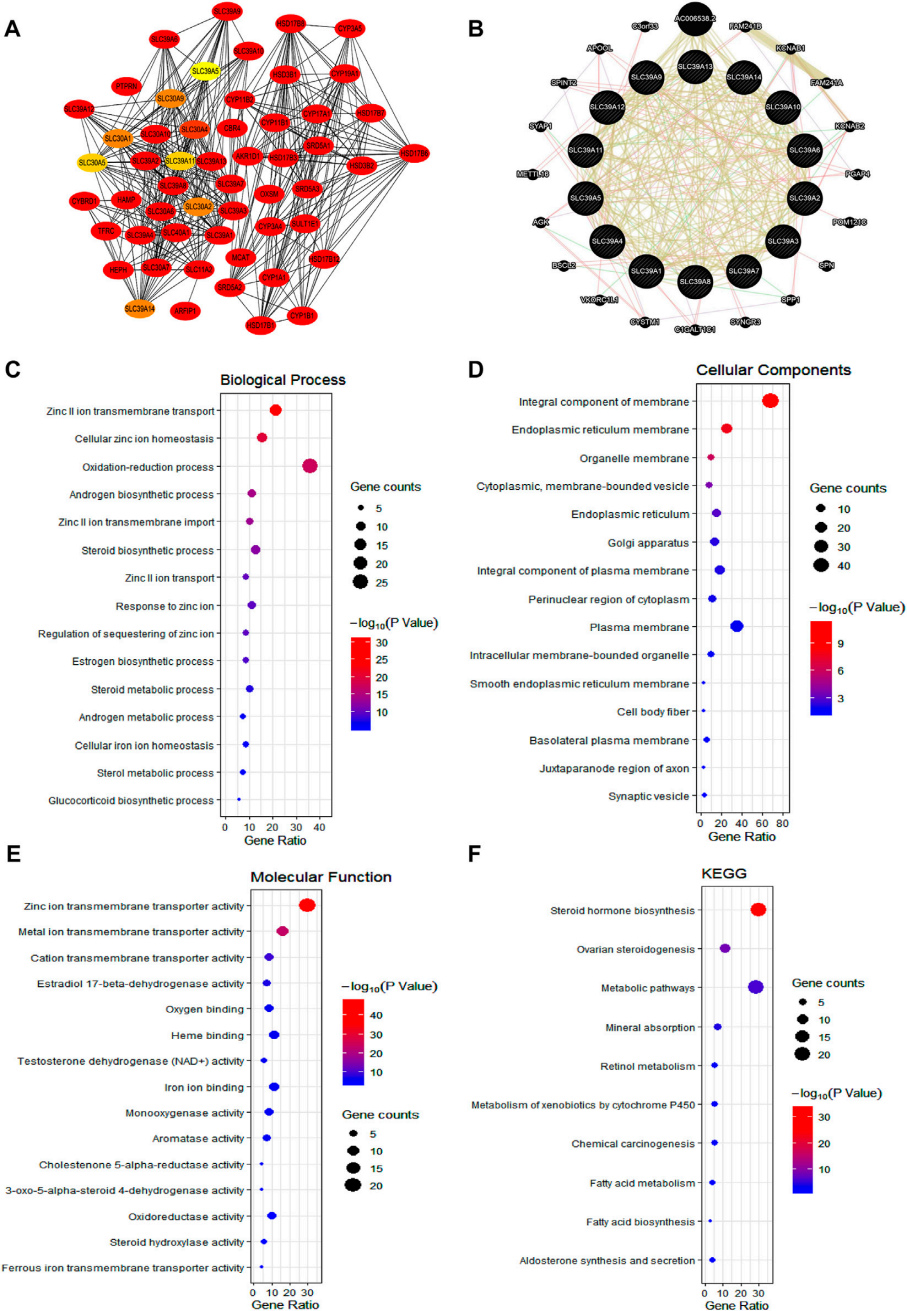
Function enrichment of the *SLC39A* family genes in pan-cancer. **(A)** Protein–protein interaction of *SLC39A* family in the STRING dataset. **(B)** Gene–gene interaction network among *SLC39A* family members in the GeneMANIA dataset. **(C)** Bubble chart showing the BP of *SLC39A* family highly correlated genes. **(D)** Bubble chart showing the CC of *SLC39A* family highly correlated genes. **(E)** Bubble chart showing the MF of *SLC39A* family highly correlated genes. **(H)** Bubble chart showing the KEGG of SLC39A family highly correlated genes.

To investigate the potential mechanism of the *SLC39A* family genes affecting the prognosis and progression of tumors, the protein–protein interactions (PPIs) of the *SLC39A* family highly related genes were conducted by using the STRING and GeneMANIA platforms. The STRING web was used to conduct the protein–protein interaction (PPI) network analysis of the *SLC39A* family genes. As expected, 54 nodes and 410 edges were obtained in the PPI network, and the 10 top-ranked node genes were *CBR4*, *MCAT*, *CYP17A1*, *HSD17B6*, *HEPH*, *SRD5A1*, *HSD17B3*, *CYP11B2*, *CYP11B1*, and *CYP19A1* (Figure 6A; Supplementary Table S5 and Supplementary Figure S1). In addition, the GeneMANIA results revealed that a total of 34 genes (including the *SLC39A* family genes) are associated with co-expression, genetic interactions, physical interactions, and shared protein domains. Among them, relationships of co-expression were predicted between *SLC39A1* and *SLC39A13*, *SLC39A1* and *SLC39A7*, *SLC39A4* and *SLC39A14*, *SLC39A5* and *SLC39A14*, *SLC39A5* and *SLC39A4*, *SLC39A6* and *SLC39A10*, and *SLC39A8* and *SLC39A14*. Genetic interactions were predicted between *SLC39A9* and *SLC39A8*, *SLC39A9* and *SLC39A3*, *SLC39A11* and *SLC39A13*, and *SLC39A11* and *SLC39A14*. Moreover, *SLC39A1* and *SLC39A2*, *SLC39A5* and *SLC39A10*, *SLC39A5* and *SLC39A6*, and *SLC39A9* and *SLC39A2* were found to share physical interactions. Most of the *SLC39A* family genes were found to share protein domains (Figure 6B).

The functional enrichment of the *SLC39A* family’s highly related genes was predicted by analyzing the GO annotation and KEGG pathway *via* the DAVID platform. According to the results (Figures 6C–F and Table 1), the function of the *SLC39A* highly related genes was enriched in the zinc II ion transmembrane transport, cellular zinc ion homeostasis, oxidation-reduction process, androgen biosynthetic process, and zinc II ion transmembrane import in biological processes (BPs). As for the cellular component (CC), the *SLC39A* family highly related genes were enriched in the integral components of the membrane, endoplasmic reticulum membrane, organelle membrane, cytoplasmic, membrane-bounded vesicle, and endoplasmic reticulum. Moreover, the *SLC39A* family influenced molecular functions through histone methyltransferase binding. With respect to the molecular function (MF), the *SLC39A* family of highly related genes was enriched in the zinc ion transmembrane transporter activity, metal ion transmembrane transporter activity, cation transmembrane transporter activity, estradiol 17-beta-dehydrogenase activity, and oxygen binding. Meanwhile, in the KEGG analysis, 10 pathways were significantly enriched, including the steroid hormone biosynthesis, ovarian steroidogenesis, metabolic pathways, mineral absorption, retinol metabolism, metabolism of xenobiotics by cytochrome P450, chemical carcinogenesis, fatty acid metabolism, fatty acid biosynthesis, and aldosterone synthesis and secretion.

**TABLE 1:** Top 10 GO and KEGG functional enrichment of SLC39A family highly related genes in pan-cancer derived from STRING, GENEMAIN, and DAVID datasets.

Category	GeneSet	Term description	%	*p*-value	FDR
BP	GO:0071577	Zinc II ion transmembrane transport	21.43	2.30E-31	8.15E-29
BP	GO:0006882	Cellular zinc ion homeostasis	15.71	1.13E-20	1.99E-18
BP	GO:0055114	Oxidation-reduction process	35.71	2.84E-18	3.35E-16
BP	GO:0006702	Androgen biosynthetic process	11.43	4.23E-15	3.74E-13
BP	GO:0071578	Zinc II ion transmembrane import	10.00	2.45E-14	1.74E-12
BP	GO:0006694	Steroid biosynthetic process	12.86	4.60E-13	2.72E-11
BP	GO:0006829	Zinc II ion transport	8.57	5.20E-11	2.63E-09
BP	GO:0010043	Response to zinc ion	11.43	9.89E-11	4.38E-09
BP	GO:0061088	Regulation of sequestering of zinc ion	8.57	1.17E-10	4.59E-09
BP	GO:0006703	Estrogen biosynthetic process	8.57	4.25E-10	1.50E-08
CC	GO:0016021	Integral component of membrane	68.57	7.68E-12	7.14E-10
CC	GO:0005789	Endoplasmic reticulum membrane	25.71	1.49E-08	6.93E-07
CC	GO:0031090	Organelle membrane	10.00	9.36E-07	2.90E-05
CC	GO:0016023	Cytoplasmic, membrane-bounded vesicle	8.57	1.76E-04	0.004101
CC	GO:0005783	Endoplasmic reticulum	15.71	0.001061	0.019736
CC	GO:0005794	Golgi apparatus	14.29	0.005088	0.078862
CC	GO:0005887	Integral component of plasma membrane	18.57	0.006431	0.085435
CC	GO:0048471	Perinuclear region of cytoplasm	11.43	0.008955	0.104104
CC	GO:0005886	Plasma membrane	35.71	0.014211	0.146847
CC	GO:0043231	Intracellular membrane-bounded organelle	10.00	0.01889	0.175682
MF	GO:0005385	Zinc ion transmembrane transporter activity	30.00	9.28E-48	1.13E-45
MF	GO:0046873	Metal ion transmembrane transporter activity	15.71	5.26E-24	3.21E-22
MF	GO:0008324	Cation transmembrane transporter activity	8.57	3.84E-10	1.56E-08
MF	GO:0004303	Estradiol 17-beta-dehydrogenase activity	7.14	4.67E-08	1.43E-06
MF	GO:0016712	Oxidoreductase activity, acting on paired donors, with incorporation or reduction of molecular oxygen, reduced flavin or flavoprotein as one donor, and incorporation of one atom of oxygen	7.14	2.99E-07	7.30E-06
MF	GO:0019825	Oxygen binding	8.57	1.14E-06	2.01E-05
MF	GO:0020037	Heme binding	11.43	1.15E-06	2.01E-05
MF	GO:0047035	Testosterone dehydrogenase (NAD+) activity	5.71	2.07E-06	3.15E-05
MF	GO:0005506	Iron ion binding	11.43	2.41E-06	3.27E-05
MF	GO:0016705	Oxidoreductase activity, acting on paired donors, with incorporation or reduction of molecular oxygen	8.57	3.02E-06	3.65E-05
KEGG	hsa00140	Steroid hormone biosynthesis	30.00	5.49E-34	1.92E-32
KEGG	hsa04913	Ovarian steroidogenesis	11.43	4.26E-09	7.45E-08
KEGG	hsa01100	Metabolic pathways	28.57	1.85E-06	2.16E-05
KEGG	hsa04978	Mineral absorption	7.14	7.39E-05	6.47E-04
KEGG	hsa00830	Retinol metabolism	5.71	0.004406	0.030844
KEGG	hsa00980	Metabolism of xenobiotics by cytochrome P450	5.71	0.006615	0.038587
KEGG	hsa05204	Chemical carcinogenesis	5.71	0.008206	0.041031
KEGG	hsa01212	Fatty acid metabolism	4.29	0.025834	0.113026
KEGG	hsa00061	Fatty acid biosynthesis	2.86	0.065993	0.233413
KEGG	hsa04925	Aldosterone synthesis and secretion	4.29	0.066689	0.233413

FDR, false discovery rate; GO, gene ontology; BP, biological processes; CC, cellular component; MF, molecular function; KEGG, Kyoto Encyclopedia of Genes and Genomes.

### The Immune Infiltration of the SLC39A Family of Genes in Pan-Cancer

**FIGURE 7:**
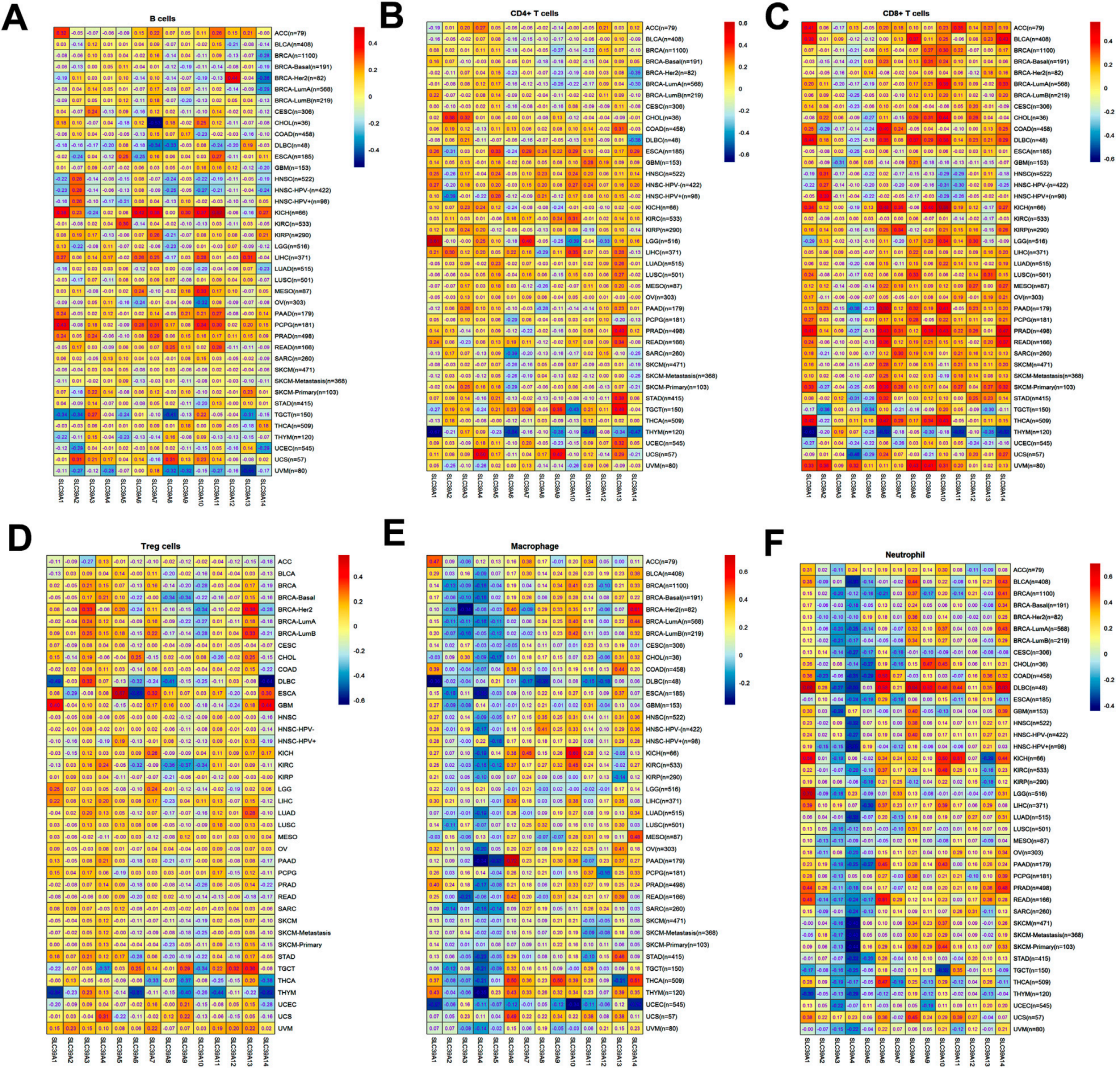
Immune cell infiltration of the *SLC39A* family genes in pan-cancer derived from the TIMER2.0 dataset. **(A)** Correlation coefficient between *SLC39A* family gene expression and B-cell infiltration score in pan-cancer. **(B)** Correlation coefficient between *SLC39A* family gene expression and CD4^+^ T-cell infiltration score in pan-cancer. **(C)** Correlation coefficient between SLC39A family gene expression and CD8^+^ T-cell infiltration score in pan-cancer. **(D)** Correlation coefficient between *SLC39A* family gene expression and Treg T-cell infiltration score in pan-cancer. **(E)** Correlation coefficient between *SLC39A* family gene expression and macrophage cell infiltration score in pan-cancer. **(F)** Correlation coefficient between *SLC39A* family gene expression and neutrophil cell infiltration score in pan-cancer. The association was generated with tumor purification adjusted.

To explore whether the *SLC39A* family genes affect the tumor immune infiltrating and microenvironment in pan-cancer, the TIMER 2.0 was used to evaluate the relationship between the *SLC39A* family gene expression levels and the infiltration of six common immune cells, including B cells, CD4^+^ T cells, CD8^+^ T cells, Treg T cells, macrophages, and neutrophils. The results confirmed a positive correlation between B-cell infiltration in ACC, KICH, KIRP, LIHC, PCPG, and PRAD but found a negative relation to B-cell immunity in COAD, DLBC, HNSC, KIRC, OV, SKCM, TGCT, TKYM, UCEC, and UVM (Figure 7A). For the CD4+ T cell, most of the *SLC39A* family members were positively related to immune infiltration in ESCA, GBM, HNSC, LIHC, and TCGT, while most of the *SLC39A* family genes were negatively correlated with the immune infiltration in BRCA and THYM (Figure 7B). For the CD8+ T cell, most of the *SLC39A* family genes were positively correlated with immune infiltration in the ACC, BLCA, DLBC, KICH, PRAD, and UVM, while most of the *SLC39A* family genes were negatively correlated with the immune infiltration in the HNSC, THYM, and UCEC (Figure 7C). Besides, a positive correlation was observed in most *SLC39A* family genes and Treg cell infiltration in TGCT and UVM, and a negative correlation was observed in the DLBC and THYM (Figure 7D). Particularly, a negative correlation was observed in most SLC39A family genes and macrophage cell infiltration in the BRCA, DLBC, UCEC, and UVM (Figure 7E). In addition, a positive correlation was observed in most of the *SLC39A* family genes and neutrophil infiltration (Figure 7F). It is noteworthy that the *SLC39A*2, *SLC39A3*, *SLC39A3*, and *SLC39A5* showed a significant negative correlation with the macrophages and neutrophil cell infiltration in most tumor types.

### Drug Sensitivity Analysis of the SLC39A Family Genes in Pan-Cancer

**FIGURE 8:**
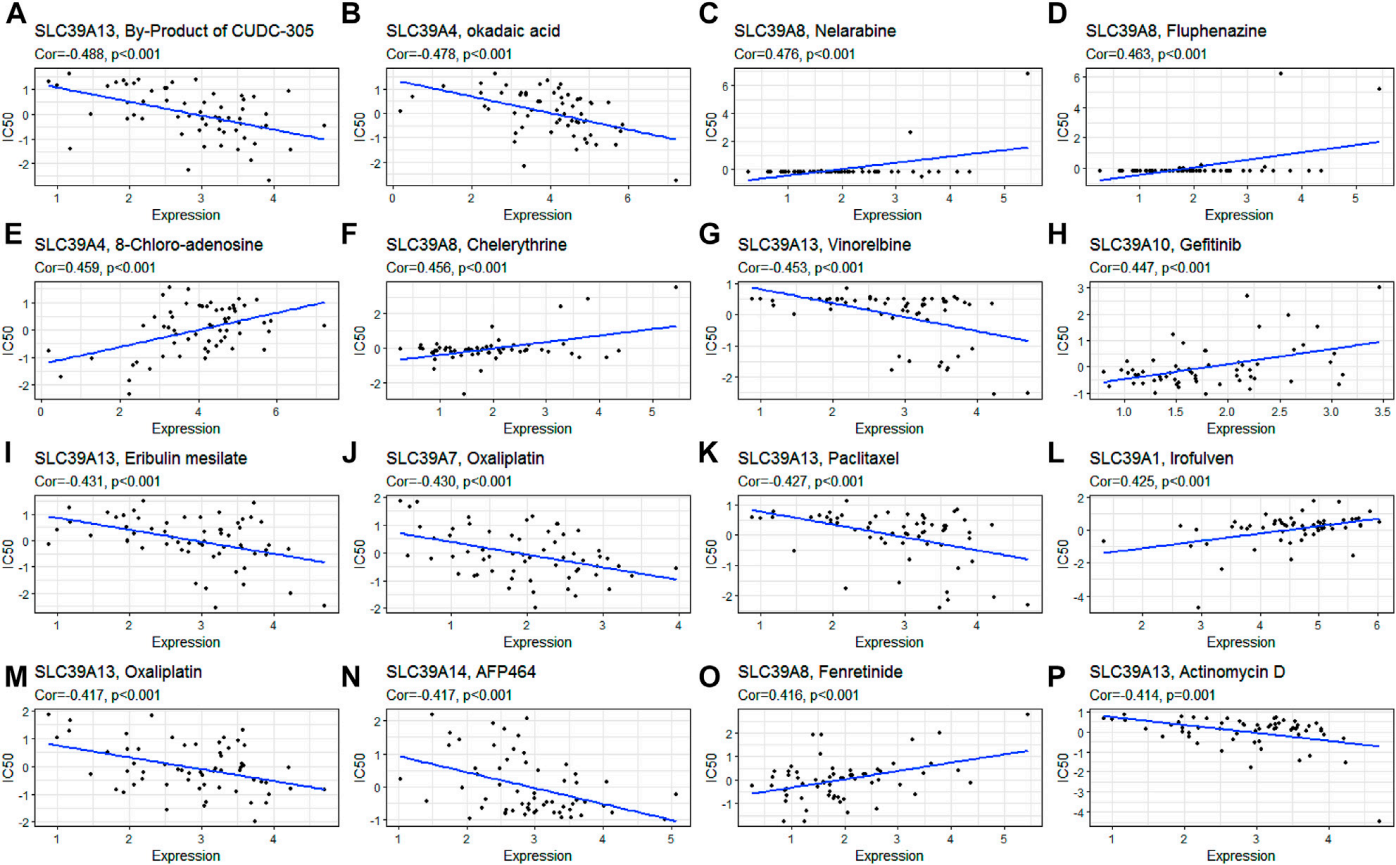
Association of the *SLC39A* family gene expression with the drug sensitivity derived from the NCI-60 cell line data. **(A)** Scatter plot of negative correlation between *SLC39A13* expression and the sensitivity of the by-product of CUDC-305. **(B)** Scatter plot of negative correlation between *SLC39A4* expression and the sensitivity of okadaic acid. **(C)** Scatter plot of positive correlation between *SLC39A8* expression and the sensitivity of nelarabine. **(D)** Scatter plot of positive correlation between *SLC39A8* expression and the sensitivity of fluphenazine. **(E)** Scatter plot of positive correlation between *SLC39A4* expression and the sensitivity of 8-chloro-adenosine. **(F)** Scatter plot of negative correlation between *SLC39A13* expression and the sensitivity of chelerythrine. **(G)** Scatter plot of positive correlation between *SLC39A4* expression and the sensitivity of vinorelbine. **(H)** Scatter plot of positive correlation between *SLC39A10* expression and the sensitivity of gefitinib. **(I)** Scatter plot of negative correlation between *SLC39A13* expression and the sensitivity of eribulin mesilate. **(J)** Scatter plot of negative correlation between *SLC39A7* expression and the sensitivity of oxaliplatin. **(K)** Scatter plot of negative correlation between *SLC39A13* expression and the sensitivity of paclitaxel. **(L)** Scatter plot of positive correlation between *SLC39A1* expression and the sensitivity of Irofulven. **(M)** Scatter plot of negative correlation between *SLC39A13* expression and the sensitivity of oxaliplatin. **(N)** Scatter plot of negative correlation between *SLC39A14* expression and the sensitivity of AFP464. **(O)** Scatter plot of positive correlation between *SLC39A8* expression and the sensitivity of fenretinide. **(P)** Scatter plot of negative correlation between *SLC39A13* expression and the sensitivity of actinomycin D. Z-score from test by Pearson’s correlation using NCI-60 cell line data.

To explore the potential sensitization or the effects of drug resistance of the *SLC39A* family genes on the drug response of different human cancer cell lines, a Pearson correlation analysis was performed between the mRNA expression of the *SLC39A* family genes in the NCI-60 cancer cell line and the drug activity of 263 antitumor drugs (Figure 8; Table 2; and Supplementary Figure S2). The results demonstrated the upregulation of *SLC39A1* expression to reduce the drug sensitivity of imisone, oxaliplatin, ifosfamide, eribulin mesylate, palbociclib, and paclitaxel but enhanced the drug sensitivity of Irofulven. An increase in the SLC39A2 expression enhanced the drug sensitivity of Isotretinoin, and the sensitivity of cladribine was found to increase by SLC39A. Various tumor cells with high expression of SLC39A4 were found to be more resistant to the okadaic acid and are more sensitive to 8-chloroadenosine and allopurinol. An increase in the SLC39A5 expression enhanced the drug sensitivity of tegafur, fluorouracil, and BML-277. Notably, the upregulation of SLC39A6 expression was found to increase the sensitivity of raloxifene and fulvestrant. High SLC39A7 expression was found to increase the drug resistance of oxaliplatin, palbociclib, dexrazoxane, entinostat, carfilzomib, epirubicin, and teniposide. However, an elevation in the *SLC39A8* gene expression was found to enhance the drug sensitivity of nelarabine, fluphenazine, chelerythrine, fenretinide, imexon, hydroxyurea, cyclophosphamide, and pipobroman. In addition, the *SLC39A10* gene expression was found to increase the drug sensitivity of gefitinib, afatinib, erlotinib, lapatinib, vandetanib, ibrutinib, and bosutinib and also increased the tolerance of cell lines to elesclomol, paclitaxel, tyrothricin, and vinorelbine. The *SLC39A12* gene expression increased the drug sensitivity of PD-98059, vemurafenib, selumetinib, hypothemycin, and dabrafenib and also increased the tolerance of the cell lines to dasatinib. The expression of SLC39A13 was found to reduce the drug sensitivity of the by-product of CUDC-305, vinorelbine, eribulin mesilate, paclitaxel, oxaliplatin, actinomycin D, nilotinib, homoharringtonine, LDK-378, vinblastine, dolastatin 10, tamoxifen, imatinib, AFP464, tanespimycin, crizotinib, palbociclib, and carfilzomib and enhance the drug sensitivity of simvastatin. At last, the expression of the *SLC39A14* gene was also found to increase the resistance of multiple drugs, including AFP464, panobinostat, cyclophosphamide, palbociclib, lificguat, and fulvestrant. On the other hand, the *SLC39A14* gene expression was found to increase the drug sensitivity of entinostat.

**TABLE 2:** Relationship between SLC39A family gene expression and drug sensitivity based on the NCI-60 cell line.

Gene	Drug	cor	*p*-value	NSC#	PubChem SID
SLC39A13	By-product of CUDC-305	−0.488	7.51E-05	761390	-
SLC39A4	Okadaic acid	−0.478	0.000113	677083	516878
SLC39A8	Nelarabine	0.476	0.000122	755985	144074932
SLC39A8	Fluphenazine	0.463	0.000194	92339	398387
SLC39A4	8-chloro-adenosine	0.459	0.000226	354258	464177
SLC39A8	Chelerythrine	0.456	0.000254	36405	544923
SLC39A13	Vinorelbine	−0.453	0.000274	760087	144076280
SLC39A10	Gefitinib	0.447	0.000338	759856	144076186
SLC39A13	Eribulin mesilate	−0.431	0.000579	707389	529374
SLC39A7	Oxaliplatin	−0.430	0.000602	266046	569872
SLC39A13	Paclitaxel	−0.427	0.000661	758645	144075668
SLC39A1	Irofulven	0.425	0.000704	683863	520035
SLC39A13	Oxaliplatin	−0.417	0.00091	266046	569872
SLC39A14	AFP464	−0.417	0.000911	710464	530822
SLC39A8	Fenretinide	0.416	0.000953	760419	144076412
SLC39A13	Actinomycin D	−0.414	0.001015	755841	144074852
SLC39A10	Afatinib	0.413	0.00103	750691	131407778
SLC39A13	Nilotinib	−0.411	0.001111	747599	91148446
SLC39A1	Imexon	−0.411	0.001115	714597	532526
SLC39A6	Raloxifene	0.411	0.00112	747974	91148450
SLC39A14	Panobinostat	−0.409	0.001172	761190	-
SLC39A13	Homoharringtonine	−0.407	0.001261	758253	-
SLC39A13	LDK-378	−0.405	0.00134	777193	-
SLC39A13	Vinblastine	−0.403	0.001413	757384	144075282
SLC39A4	Allopurinol	0.403	0.001424	1,390	68199
SLC39A10	Erlotinib	0.397	0.001684	718781	534851
SLC39A1	Oxaliplatin	−0.394	0.001825	266046	569872
SLC39A10	Lapatinib	0.393	0.00192	745750	91147938
SLC39A8	Imexon	0.391	0.001991	714597	532526
SLC39A1	Ifosfamide	−0.390	0.002069	109724	301170
SLC39A7	Palbociclib	−0.389	0.002157	758247	-
SLC39A12	PD-98059	0.381	0.002652	679828	518213
SLC39A13	Dolastatin 10	−0.380	0.002743	376128	469333
SLC39A13	Tamoxifen	−0.378	0.002902	180973	447264
SLC39A10	Vandetanib	0.377	0.002996	760766	131408693
SLC39A12	Vemurafenib	0.375	0.003169	761431	131408691
SLC39A6	Fulvestrant	0.374	0.003201	719276	534986
SLC39A10	Elesclomol	−0.372	0.003428	174939	445356
SLC39A10	Ibrutinib	0.370	0.003626	761910	-
SLC39A10	Bosutinib	0.370	0.003646	765694	-
SLC39A12	Selumetinib	0.366	0.003978	741078	91146061
SLC39A7	Dexrazoxane	−0.366	0.004008	169780	442425
SLC39A13	Imatinib	−0.366	0.004011	743414	91146949
SLC39A5	Tegafur	0.366	0.004025	148958	430704
SLC39A13	AFP464	−0.365	0.004084	710464	530822
SLC39A13	Tanespimycin	−0.364	0.004255	330507	574817
SLC39A10	Paclitaxel	−0.361	0.004571	758645	144075668
SLC39A1	Eribulin mesilate	−0.358	0.004949	707389	529374
SLC39A13	Crizotinib	−0.358	0.005009	756645	131408690
SLC39A5	Fluorouracil	0.357	0.005048	757036	144075048
SLC39A13	Palbociclib	−0.357	0.005157	758247	-
SLC39A8	Hydroxyurea	0.355	0.00534	32065	90752
SLC39A1	Palbociclib	−0.354	0.005531	758247	-
SLC39A14	Cyclophosphamide	−0.354	0.005552	26271	87150
SLC39A10	Tyrothricin	−0.354	0.005564	757363	144075261
SLC39A5	BML-277	0.353	0.005734	741899	91146360
SLC39A7	Entinostat	−0.351	0.00593	706995	529250
SLC39A8	Cyclophosphamide	0.351	0.006	26271	87150
SLC39A10	Vinorelbine	−0.348	0.006518	760087	144076280
SLC39A13	Carfilzomib	−0.347	0.006546	758252	-
SLC39A14	Palbociclib	−0.345	0.006897	758247	-
SLC39A13	Simvastatin	0.345	0.006942	758706	144075729
SLC39A14	Lificguat	−0.344	0.007171	728165	48427734
SLC39A12	Hypothemycin	0.343	0.007328	354462	576295
SLC39A7	Carfilzomib	−0.342	0.007394	758252	-
SLC39A12	Dabrafenib	0.340	0.007788	764134	-
SLC39A3	Cladribine	0.339	0.008008	105014	405818
SLC39A14	Fulvestrant	−0.339	0.008077	719276	534986
SLC39A2	Isotretinoin	0.338	0.008346	122758	416403
SLC39A14	Zoledronate	0.336	0.008774	721517	536160
SLC39A1	Paclitaxel	−0.333	0.009309	758645	144075668
SLC39A7	Epirubicin	−0.333	0.009424	759195	144075933
SLC39A14	Entinostat	−0.332	0.009669	706995	529250
SLC39A7	Teniposide	−0.331	0.009681	758667	144075690
SLC39A12	Dasatinib	−0.331	0.009702	759877	144076207
SLC39A8	Pipobroman	0.331	0.009745	25154	86412

cor, correlation coefficient; NSC#, Cancer Chemotherapy National Service Center number; PubChem SID, PubChem Substance IDs.

## Discussion

Zinc, as an essential trace element, participates in various physiological events, such as growth, differentiation, development, immunity, apoptosis, and other physiological processes (Kimura and Kambe, 2016). Previous studies have reported that zinc is required for over 300 enzymes’ activity and 2,000 transcription factors to work (Prasad, 2012). Thus, zinc metabolism and homeostasis regulate the normal cell functions in a complex manner. Aberrant Zn transporters have been reported to contribute to specific diseases, including endocrine diseases, neurodegenerative diseases, metabolic diseases, cardiovascular diseases, immune deficiencies, and cancers (Prasad, 2012; Kambe et al., 2014). In particular, current evidence suggests that zinc deficiency and dysregulation of the zinc metabolism are risk factors for tumorigenesis, and zinc is considered a tumor-suppressive agent and a potential tumor treatment target (Grattan and Freake, 2012; Pan et al., 2017; Zhang et al., 2021). In addition, the two groups of zinc transporters, the ZnT transporter (SLC30A) and the ZIP channel (SLC39A), exert strict control over the concentration of zinc in cells, and the SLC39A are known to operate in the influx of zinc across the cytoplasm from the extracellular environment into the cytosol (Hojyo and Fukada, 2016; Pan et al., 2017; Brito et al., 2020). The available data suggest that the mutation or functional change of the *SLC39A* family of genes develops various diseases such as the tumors of the digestive system, urinary system, and reproductive tract (Prasad, 2012; Hoang et al., 2016; To et al., 2020). Interestingly, the SLC39A family gene knockout animals have revealed many unique phenotypes and the possibility of the clinical targeted application and the possibility of discovery and development of the SLC39A family inhibitors as anticancer drugs and modulators regulating the sensitivity or resistance of chemotherapeutic drugs (Geng et al., 2018; Hu, 2020; Mohammadinejad et al., 2020; Cheng et al., 2021). However, the precise function of the *SLC39A* family genes in pan-cancer has not been comprehensively determined.

In the current study, compared to the normal control group, the expression levels of most of the *SLC39* family genes in the tumor tissues were found to be significantly upregulated. The high expression of SLC39A6 is a dependable marker for breast cancer (luminal A subtype), and elevated *SLC39A10* mRNA levels were evident in the cancer cell lines of the highly aggressive breast cancer (Kagara et al., 2007; Hogstrand et al., 2013). Cheng et al. (2017) have found the ESCC tissues to possess an increased mRNA expression level of *SLC39A6* compared to the non-tumor tissues. Studies by Li et al. (2007) reported that compared to the human pancreatic ductal epithelium (HPDE) cells, the expression of the *SLC39A4* mRNA was significantly increased in the human pancreatic cancer cells. In addition, a bioinformatics study also found increased expression levels of the *SLC39A* family genes with significant upregulation in the breast cancer, gastric cancer, and lung cancer tissues compared to the normal breast tissues (Ding et al., 2019; Liu et al., 2020; Zhou et al., 2021). There are not many studies on the genetic mutations of the SLC39A family in tumor tissues. Our study reported that the mutation frequencies of the *SLC39A4* and *SLC39A1* were the highest among all the members and were 6 and 4%, respectively. The overall mutation frequency of the *SLC39A* family genes was in the general range of 0.8–6.0% in pan-cancer. Moreover, the mutations in the *SLC39A* family genes were found to have a significant impact on the DSS, PFS, and DFS of the malignant tumor.

For prognostic analysis, the expression of SLC39A family genes was found to be significantly related to the OS and RFS in multiple types using the KM-plotter database. Most *SLC39A* family genes showed protective effects in BLCA, CESC, HNSC, BRCA, ESCA, and OV. Simultaneously, the PrognoScan results indicated that the *SLC39A* family gene expression levels were significantly correlated with the prognosis of the colorectal, breast, bladder, lung, ovarian, blood, brain, skin, eye, soft tissue, prostate, and head and neck cancers. Our findings were consistent with those of the previous study, in that high mRNA expression levels of *SLC39A6* and *SLC39A14* indicated favorable OS, but upregulated *SLC39A2-5*, *SLC39A7*, and *SLC39A12-13* were associated with poor OS in the patients with breast carcinoma (Liu et al., 2020). Higher expression of SLC39A1, 5–7, and 9 indicated better OS, FPS, and PPS, and increased SLC39A2–4, 8, and SLC39A10 expression indicated poor OS, FP, and PPS in the patients with gastric cancer (Ding et al., 2019). In addition, an increase in the SLC39A7 expression was related to better OS, while the upregulated level of SLC39A3 and SLC39A4 were associated with inferior OS in patients with LUSC (Zhou et al., 2021). Consistent with previous research, the GO function enrichment indicated the SLC39A family genes and their highly related genes to contribute to zinc transport– and homeostasis-related biological processes, such as zinc II ion transmembrane transport, cellular zinc ion homeostasis, oxidation-reduction, androgen biosynthesis, and zinc II ion transmembrane import. The KEGG analysis showed *SLC39A* family genes to be involved in the hormone regulation, metabolic pathways, mineral absorption, and chemical carcinogenesis pathways (Ding et al., 2019; Zhou et al., 2021). Therefore, the prognostic effect of the SLC39A family genes can be speculated to be closely related to zinc transfer, metabolism, and function (Guo and He, 2020).

Increasing studies have shown that zinc is involved in a variety of important functions of immune cell activation and initiation of immune response in the process of innate immunity and adaptive immunity; thus, zinc deficiency can lead to immune dysfunction (Bin et al., 2018). Given the close relationship between the SLC39A family and zinc transport, the regulatory relationship between the *SLC39A* family genes and immune infiltration deserves more attention. SLC39A6 and SLC39A10 are the first zinc transporters reported to regulate immune cell functions in mammals. Subsequent studies have confirmed the role of SLC39A8 in regulating various immune cells and playing an irreplaceable role in the process of innate immunity (Kitamura et al., 2006; Liu et al., 2013). The study of Hojyo et al. (2014) reported the SLC39A10 expression to be upregulated in pro-B lymphocytes and SLC39A10 to participate in B-cell immunity by leading the homeostasis and the function of the B cells. Our findings suggested a significant correlation between the *SLC39A* family gene expression and B-cell infiltration in broad cancer types and CD4^+^ T cells, CD8^+^ T cells, Treg T cells, macrophages, and neutrophils in specific tumors. These results provide new possibilities for immunotherapy to improve the prognosis by modulating the *SLC39A* family genes on the tumors or immune cells. Presently, there have been a few attempts of transformational application research using the SLC39A family to treat or alleviate diseases in animals or cell models. One study showed that in an *in vivo* xenograft model, the overexpression of the SLC39A1 leads to an increased zinc uptake, reducing the tumor growth (Golovine et al., 2008). Utilizing the characteristics of the zinc SLC39A6 transporter widely expressed in all breast cancer subtypes, Seattle Genetics has designed and constructed a new antibody–drug conjugate called SGN-LIV1A to treat metastatic breast cancer through the targeted regulation of SLC39A6 (Sun et al., 2011). In our study, the Pearson correlation analysis was performed between the mRNA expression of the *SLC39A* family genes in the NCI-60 cancer cell line and the drug activity of 263 antitumor drugs. The results showed that SLC39A family genes are significantly related to the sensitivity or resistance of 63 antitumor drugs in a variety of tumor cell lines. Among them, SLC39A13 and the by-product of the CUDC-305, SLC39A4, okadaic acid, SLC39A8, and nelarabine are the three most likely connections. Based on these results, the detection and targeted regulation of the expression of the SLC39A family gene have been found to have special potential value for the clinical selection of antitumor drugs.

Despite being the first one to perform a multidimensional and multi-omics analysis of the *SLC39A* family genes in pan-cancer, this study has some shortcomings worth considering. First, the bioinformatics analysis was carried out through multiple online big data databases, and further *in vitro* and *in vivo* experiments are required to verify the prediction results. Second, multiple databases are not completely consistent in the expression and survival prognosis of the *SLC39A* family genes in certain tumors. Large samples, different populations, and multicenter clinical studies need further clarification. Third, although we have confirmed that the *SLC39A* family gene expression was significantly related to the immune infiltration and survival outcome of a variety of tumors, the causal relationship between the immune infiltration and prognosis remains elusive. Fourth, analyzing the level of immune infiltrating cells at the tumor tissue level may be error-prone, and hence, single-cell sequencing may be required for further exploration.

## Conclusion

This pan-cancer study performed a comprehensive and systematic investigation of the expression patterns, genetic mutation, prognostic value, function enrichment, immune infiltrating, and potential therapeutic targets of the *SLC39A* family of genes. Our results proved that most of the *SLC39* family genes’ expression was significantly increased in the tumor tissues and was associated with clinical prognosis in pan-cancer. Moreover, the *SLC39A* family gene expression was significantly related to the immune cell infiltration levels of six types of immune cells and contributed to the sensitivity or resistance of the drugs in specific types of tumors. Thus, we concluded that the *SLC39A* of family genes may be crucial for tumorigenesis, the tumor microenvironment, and drug sensitivity, providing novel ideas to develop new targeted therapy for malignant tumors.

## Data Availability

The datasets presented in this study can be found in online repositories. The names of the repository/repositories and accession number(s) can be found in the article/Supplementary Material.
