# Supplementary material for: Pan-Cancer Analysis of the Solute Carrier Family 39 Genes in Relation to Oncogenic, Immune Infiltrating, and Therapeutic Targets
Source: Front Genet. 2021 Dec 2;12:757582. doi: 10.3389/fgene.2021.757582 (PMC8675640; doi:10.3389/fgene.2021.757582)

Table S1. The average mRNA expression (TPM) of SCL39A family genes family genes in pan-cancer and their normal controls in the GEPIA match TCGA normal and GTEx data.

| cancer | SCL39A1 | SCL39A2 | SCL39A3 | SCL39A4 | SCL39A5 | SCL39A6 | SCL39A7 | SCL39A8 | SCL39A9 | SCL39A10 | SCL39A11 | SCL39A12 | SCL39A13 | SCL39A14 |
| --- | --- | --- | --- | --- | --- | --- | --- | --- | --- | --- | --- | --- | --- | --- |
| ACC | 5.8 | 0.1 | 5.7 | 4.4 | 0.2 | 4.2 | 6.6 | 3 | 4.6 | 3.4 | 3 | 0 | 4.9 | 5.4 |
| BLCA | 6.4 | 1.3 | 5.1 | 6.5 | 0.2 | 5.2 | 7.1 | 2.8 | 4.5 | 2.7 | 4.7 | 0 | 4.7 | 3.1 |
| BRCA | 6.8 | 0.5 | 5.3 | 5.6 | 0.2 | 8 | 7.1 | 3.7 | 5.5 | 3.6 | 5.5 | 0 | 4.8 | 4.2 |
| CESC | 6.6 | 1.8 | 5 | 5.9 | 0.2 | 4.9 | 6.7 | 3.2 | 4.6 | 2.6 | 4.3 | 0 | 4.1 | 3.4 |
| CHOL | 7.3 | 0.1 | 4.9 | 5.5 | 3.6 | 5.2 | 7 | 2.1 | 4.7 | 3.2 | 4.6 | 0 | 5.2 | 5.4 |
| COAD | 6.2 | 0.6 | 4.9 | 7.1 | 5.8 | 4.1 | 6.8 | 4.7 | 4.7 | 3.5 | 4.8 | 0 | 4.3 | 5.4 |
| DLBC | 5.5 | 0 | 4.5 | 5.2 | 0.1 | 3.4 | 5.9 | 4.5 | 3.9 | 1.9 | 4.2 | 0 | 4 | 3 |
| ESCA | 6.2 | 1 | 4.4 | 6.4 | 0.6 | 5 | 5.7 | 3.4 | 5.2 | 3.4 | 4.2 | 0 | 4.7 | 5.3 |
| GBM | 6.7 | 0.1 | 5.8 | 3.6 | 0.2 | 5.5 | 6.6 | 3.9 | 4.8 | 4.1 | 3.5 | 1.9 | 4.9 | 5 |
| HNSC | 6.4 | 2.7 | 4.8 | 6 | 0.1 | 5.5 | 6.6 | 2.8 | 4.8 | 2.5 | 4.4 | 0 | 4.4 | 4.3 |
| KICH | 4.9 | 0.2 | 5.3 | 4.8 | 0.1 | 3.9 | 6.1 | 2.6 | 5 | 1.5 | 3.3 | 0 | 6.9 | 3 |
| KIRC | 6.8 | 0 | 4.2 | 4.4 | 5.3 | 4.9 | 6.1 | 4.1 | 4.9 | 3.5 | 3.6 | 0 | 5.1 | 6.1 |
| KIRP | 6.9 | 0 | 4.8 | 5.7 | 5.5 | 4.3 | 6.2 | 4.4 | 4.7 | 3.3 | 4.3 | 0 | 6 | 3.8 |
| LAML | 4.8 | 0.1 | 6 | 4.4 | 0.6 | 4.2 | 4.5 | 4.6 | 5.3 | 4.3 | 5 | 0 | 4 | 2.9 |
| LGG | 5.7 | 0.1 | 5.6 | 2.5 | 0.3 | 5.7 | 6.3 | 2.9 | 4.9 | 4.5 | 4.2 | 2.1 | 4.9 | 4.6 |
| LIHC | 6.2 | 0 | 4.9 | 3.8 | 5.7 | 3 | 6.8 | 3.3 | 4.5 | 1.4 | 4.4 | 0 | 3.9 | 5.8 |
| LUAD | 6.8 | 0.1 | 4.9 | 6.4 | 0.2 | 4.8 | 7.2 | 5.1 | 4.8 | 3.4 | 5 | 0 | 5 | 3.6 |
| LUSC | 6.5 | 1 | 5 | 5.9 | 0.1 | 5.4 | 6.7 | 3.5 | 4.9 | 3.3 | 4.8 | 0 | 4.6 | 4.3 |
| MESO | 7 | 0.1 | 4.9 | 4.2 | 0.2 | 4.8 | 7.2 | 6.4 | 4.5 | 3.9 | 4.2 | 0 | 5.7 | 4.5 |
| OV | 6.4 | 0.1 | 4.6 | 7.4 | 0.3 | 4.1 | 5.6 | 3.5 | 4.2 | 3.8 | 3.7 | 0 | 4.9 | 4 |
| PAAD | 6.7 | 0.1 | 4.9 | 6.3 | 2.8 | 4.4 | 7 | 3.2 | 4.8 | 3.2 | 4.8 | 0 | 5.3 | 5.3 |
| PCPG | 5.4 | 0.1 | 5.5 | 3.9 | 0.2 | 6 | 7.2 | 1.9 | 4.8 | 2.7 | 2.7 | 0.1 | 5.6 | 4.5 |
| PRAD | 6.2 | 0.2 | 5.4 | 5.1 | 0.2 | 7.2 | 8 | 3.4 | 5.2 | 4 | 3.6 | 0 | 4.7 | 3.5 |
| READ | 6.2 | 0.4 | 4.8 | 6.9 | 5.8 | 3.9 | 6.8 | 4.7 | 4.7 | 3.7 | 4.9 | 0 | 4.2 | 5.4 |
| SARC | 6.6 | 0.1 | 5.4 | 4 | 0.2 | 4.8 | 7.2 | 3.7 | 4.5 | 2.9 | 3.7 | 0 | 5.9 | 4.7 |
| SKCM | 6.8 | 0.1 | 5.5 | 5.7 | 0.1 | 6.1 | 7.1 | 3.3 | 4.7 | 3.2 | 4.1 | 0 | 5.4 | 4.9 |
| STAD | 6.1 | 0 | 4.5 | 6.8 | 3.6 | 4.2 | 5.9 | 3.2 | 4.9 | 3.1 | 4.2 | 0 | 4.6 | 6.1 |
| TGCT | 6.4 | 0.1 | 5.1 | 5.2 | 0.4 | 3.5 | 6.7 | 3.1 | 3.8 | 4.1 | 3.5 | 0 | 3.9 | 4.4 |
| THCA | 6.3 | 0 | 5 | 5.4 | 0.3 | 5 | 6.8 | 3.4 | 4.5 | 5.1 | 4.2 | 0 | 5 | 4.9 |
| THYM | 5.5 | 1 | 4.9 | 5 | 0.2 | 3.7 | 6 | 3.6 | 4.4 | 3.1 | 2.6 | 0 | 4.6 | 2.1 |
| UCEC | 6.5 | 0.3 | 5 | 6.6 | 0.4 | 4.8 | 7.3 | 3.4 | 4.6 | 3.3 | 4.1 | 0 | 4.6 | 3.5 |
| UCS | 6.3 | 0.3 | 4.5 | 6.2 | 0.4 | 4.8 | 7.4 | 3.1 | 4.4 | 3.4 | 3.7 | 0 | 5 | 3.5 |
| UVM | 6.1 | 0 | 5.3 | 6.3 | 0.1 | 5.4 | 6.6 | 1.1 | 4 | 1.7 | 3.1 | 0 | 5.2 | 3.2 |

Table S2. The HR and 95% confidence interval of the OS of the SCL39A gene family in pan-cancer derived from KM-plotter dataset.

|  | SCL39A1 | | | | SCL39A2 | | | | SCL39A3 | | | | SCL39A4 | | | | SCL39A5 | | | | SCL39A6 | | | | SCL39A7 | | | |
| --- | --- | --- | --- | --- | --- | --- | --- | --- | --- | --- | --- | --- | --- | --- | --- | --- | --- | --- | --- | --- | --- | --- | --- | --- | --- | --- | --- | --- |
|  | HR | Low | High | P-value | HR | Low | High | P-value | HR | Low | High | P-value | HR | Low | High | P-value | HR | Low | High | P-value | HR | Low | High | P-value | HR | Low | High | P-value |
| BLCA | 1.34 | 0.98 | 1.82 | 0.07 | 1.41 | 1.03 | 1.93 | 0.03 | 0.70 | 0.52 | 0.96 | 0.02 | 1.22 | 0.86 | 1.71 | 0.26 | 0.57 | 0.43 | 0.77 | 0.00 | 1.35 | 0.94 | 1.92 | 0.10 | 1.38 | 0.94 | 2.01 | 0.10 |
| BRCA | 0.70 | 0.49 | 0.99 | 0.04 | 0.70 | 0.50 | 1.00 | 0.05 | 0.79 | 0.56 | 1.12 | 0.19 | 0.80 | 0.56 | 1.12 | 0.19 | 0.71 | 0.51 | 1.00 | 0.05 | 0.76 | 0.52 | 1.05 | 0.09 | 1.38 | 1.00 | 1.91 | 0.05 |
| CESC | 2.37 | 1.44 | 3.91 | 0.00 | 0.62 | 0.38 | 1.00 | 0.05 | 0.51 | 0.29 | 0.88 | 0.02 | 2.08 | 1.28 | 3.37 | 0.00 | 0.67 | 0.41 | 1.08 | 0.10 | 1.73 | 1.07 | 2.80 | 0.02 | 1.45 | 0.90 | 2.32 | 0.12 |
| ESCA | 0.65 | 0.29 | 1.50 | 0.31 | 1.52 | 0.68 | 3.43 | 0.31 | 1.53 | 0.67 | 3.52 | 0.31 | 0.63 | 0.24 | 1.61 | 0.33 | 0.42 | 0.15 | 1.14 | 0.08 | 1.56 | 0.67 | 3.59 | 0.30 | 0.41 | 0.18 | 0.96 | 0.03 |
| HNSC | 1.92 | 1.39 | 2.67 | 0.00 | 1.17 | 0.89 | 1.53 | 0.27 | 0.72 | 0.55 | 0.95 | 0.02 | 1.17 | 0.85 | 1.60 | 0.33 | 0.82 | 0.63 | 1.08 | 0.15 | 0.66 | 0.49 | 0.88 | 0.00 | 0.84 | 0.60 | 1.16 | 0.28 |
| KIRC | 1.35 | 0.97 | 1.86 | 0.07 | 0.73 | 0.53 | 0.99 | 0.05 | 1.81 | 1.26 | 2.58 | 0.00 | 0.78 | 0.57 | 1.08 | 0.14 | 0.45 | 0.33 | 0.61 | 0.00 | 0.82 | 0.60 | 1.10 | 0.18 | 0.70 | 0.52 | 0.95 | 0.02 |
| KIRP | 2.32 | 1.28 | 4.20 | 0.00 | 1.96 | 1.08 | 3.54 | 0.02 | 1.44 | 0.76 | 2.71 | 0.26 | 0.59 | 0.31 | 1.13 | 0.11 | 0.46 | 0.25 | 0.87 | 0.02 | 1.48 | 0.79 | 2.80 | 0.22 | 2.28 | 1.25 | 4.16 | 0.01 |
| LIHC | 2.45 | 1.71 | 3.50 | 0.00 | 0.68 | 0.47 | 0.98 | 0.04 | 1.57 | 1.11 | 2.23 | 0.01 | 1.34 | 0.94 | 1.91 | 0.11 | 0.77 | 0.53 | 1.11 | 0.16 | 1.85 | 1.31 | 2.62 | 0.00 | 2.18 | 1.41 | 3.38 | 0.00 |
| LUAD | 1.54 | 1.07 | 2.22 | 0.02 | 1.30 | 0.93 | 1.81 | 0.12 | 0.86 | 0.64 | 1.16 | 0.33 | 1.40 | 0.99 | 1.98 | 0.06 | 0.73 | 0.53 | 0.99 | 0.04 | 1.33 | 0.98 | 1.78 | 0.06 | 1.19 | 0.88 | 1.61 | 0.27 |
| LUSC | 1.19 | 0.91 | 1.56 | 0.20 | 1.30 | 0.97 | 1.75 | 0.08 | 0.74 | 0.55 | 1.00 | 0.05 | 1.23 | 0.94 | 1.61 | 0.14 | 1.21 | 0.90 | 1.62 | 0.20 | 0.71 | 0.53 | 0.95 | 0.02 | 0.78 | 0.59 | 1.03 | 0.08 |
| OV | 1.13 | 0.86 | 1.49 | 0.39 | 0.65 | 0.47 | 0.88 | 0.01 | 0.76 | 0.58 | 0.99 | 0.04 | 0.75 | 0.58 | 0.97 | 0.03 | 0.83 | 0.61 | 1.11 | 0.21 | 0.81 | 0.62 | 1.07 | 0.13 | 0.70 | 0.54 | 0.92 | 0.01 |
| PAAD | 2.01 | 1.17 | 3.45 | 0.01 | 1.43 | 0.90 | 2.26 | 0.12 | 0.54 | 0.36 | 0.82 | 0.00 | 1.67 | 1.10 | 2.54 | 0.01 | 1.52 | 0.96 | 2.42 | 0.07 | 0.80 | 0.51 | 1.25 | 0.32 | 1.25 | 0.80 | 1.94 | 0.33 |
| PCPG | 2.95 | 0.59 | 14.66 | 0.17 | 17.09 | 1.99 | 146.80 | 0.00 | 4.11 | 0.73 | 23.16 | 0.09 | 0.00 | 0.00 | inf | 0.04 | 0.46 | 0.09 | 2.28 | 0.33 |  | 0.00 | inf | 0.09 | 0.42 | 0.08 | 2.20 | 0.29 |
| READ | 2.46 | 1.10 | 5.48 | 0.02 | 1.51 | 0.69 | 3.28 | 0.30 | 1.97 | 0.88 | 4.41 | 0.09 | 2.06 | 0.93 | 4.55 | 0.07 | 0.53 | 0.22 | 1.32 | 0.17 | 0.49 | 0.22 | 1.05 | 0.06 | 0.56 | 0.26 | 1.22 | 0.14 |
| SARC | 1.32 | 0.84 | 2.08 | 0.23 | 0.36 | 0.20 | 0.65 | 0.00 | 0.64 | 0.41 | 0.99 | 0.04 | 1.51 | 0.98 | 2.34 | 0.06 | 1.43 | 0.89 | 2.30 | 0.14 | 1.69 | 1.12 | 2.54 | 0.01 | 1.80 | 1.18 | 2.75 | 0.01 |
| STAD | 0.68 | 0.49 | 0.94 | 0.02 | 0.60 | 0.42 | 0.87 | 0.01 | 0.66 | 0.47 | 0.09 | 0.01 | 1.45 | 1.03 | 2.03 | 0.03 | 0.81 | 0.55 | 1.20 | 0.29 | 0.82 | 0.59 | 1.14 | 0.24 | 0.88 | 0.63 | 1.24 | 0.47 |
| TGCT | 0.00 | 0.00 | inf | 0.12 | 5.40 | 0.49 | 59.57 | 0.12 | 0.16 | 0.02 | 1.51 | 0.07 | 2.28 | 0.32 | 16.32 | 0.40 | 2.74 | 0.39 | 19.46 | 0.29 | 0.00 | 0.00 | inf | 0.04 | 2.84 | 0.40 | 20.16 | 0.28 |
| THCA | 4.64 | 1.22 | 17.70 | 0.01 | 2.47 | 0.51 | 11.95 | 0.24 | 0.15 | 0.03 | 0.71 | 0.01 | 0.26 | 0.07 | 0.96 | 0.03 | 1.92 | 0.48 | 7.72 | 0.35 | 3.28 | 0.85 | 12.63 | 0.07 | 0.16 | 0.02 | 1.29 | 0.05 |
| THYM | 2.00 | 0.75 | 5.39 | 0.16 | 0.37 | 0.14 | 1.01 | 0.04 | 0.19 | 0.03 | 1.46 | 0.08 | 9.20 | 1.21 | 69.64 | 0.01 | 0.34 | 0.13 | 0.92 | 0.03 | 2.73 | 0.62 | 12.02 | 0.17 | 3.82 | 1.32 | 11.02 | 0.01 |
| UCEC | 0.79 | 0.48 | 1.28 | 0.34 | 0.76 | 0.51 | 1.16 | 0.20 | 0.31 | 0.16 | 0.60 | 0.00 | 1.50 | 0.99 | 2.27 | 0.05 | 2.80 | 1.82 | 4.33 | 0.00 | 0.50 | 0.31 | 0.82 | 0.00 | 0.65 | 0.43 | 0.98 | 0.04 |
|  | SCL39A8 | | | | SCL39A9 | | | | SCL39A10 | | | | SCL39A11 | | | | SCL39A12 | | | | SCL39A13 | | | | SCL39A14 | | | |
|  | HR | Low | High | P-value | HR | Low | High | P-value | HR | Low | High | P-value | HR | Low | High | P-value | HR | Low | High | P-value | HR | Low | High | P-value | HR | Low | High | P-value |
| BLCA | 1.49 | 1.11 | 2.00 | 0.01 | 1.44 | 1.08 | 1.94 | 0.01 | 1.18 | 0.87 | 1.58 | 0.29 | 0.71 | 0.51 | 1.00 | 0.05 | 0.83 | 0.61 | 1.12 | 0.21 | 1.13 | 0.84 | 1.52 | 0.42 | 1.70 | 1.24 | 2.34 | 0.00 |
| BRCA | 0.59 | 0.42 | 0.83 | 0.00 | 1.25 | 0.86 | 1.82 | 0.24 | 0.79 | 0.57 | 1.09 | 0.14 | 1.35 | 0.96 | 1.89 | 0.08 | 0.61 | 0.44 | 0.84 | 0.00 | 0.76 | 0.54 | 1.07 | 0.12 | 1.25 | 0.91 | 1.72 | 0.18 |
| CESC | 1.84 | 1.15 | 2.95 | 0.01 | 1.53 | 0.93 | 2.54 | 0.09 | 2.02 | 1.24 | 3.28 | 0.00 | 1.35 | 0.78 | 2.34 | 0.28 | 1.62 | 1.01 | 2.58 | 0.04 | 1.82 | 1.10 | 3.00 | 0.02 | 1.89 | 1.18 | 3.02 | 0.01 |
| ESCA | 0.40 | 0.12 | 1.33 | 0.12 | 0.25 | 0.08 | 0.84 | 0.02 | 0.42 | 0.15 | 1.12 | 0.07 | 1.51 | 0.67 | 3.40 | 0.32 | 1.85 | 0.72 | 4.78 | 0.20 | 1.55 | 0.62 | 3.83 | 0.34 | 0.27 | 0.11 | 0.67 | 0.00 |
| HNSC | 1.27 | 0.94 | 1.72 | 0.12 | 1.26 | 0.95 | 1.68 | 0.10 | 1.34 | 0.96 | 1.87 | 0.09 | 1.41 | 1.07 | 1.87 | 0.02 | 1.38 | 1.01 | 1.89 | 0.04 | 1.34 | 1.01 | 1.79 | 0.04 | 1.45 | 1.09 | 1.91 | 0.01 |
| KIRC | 0.48 | 0.35 | 0.64 | 0.00 | 0.37 | 0.25 | 0.55 | 0.00 | 0.69 | 0.51 | 0.94 | 0.02 | 1.82 | 1.34 | 2.48 | 0.00 | 1.57 | 1.17 | 2.12 | 0.00 | 1.62 | 1.20 | 2.18 | 0.00 | 0.83 | 0.60 | 1.15 | 0.25 |
| KIRP | 0.30 | 0.16 | 0.53 | 0.00 | 0.70 | 0.39 | 1.26 | 0.23 | 2.51 | 1.38 | 4.55 | 0.00 | 2.29 | 1.26 | 4.13 | 0.00 | 0.68 | 0.37 | 1.23 | 0.20 | 0.44 | 0.24 | 0.79 | 0.00 | 2.58 | 1.41 | 4.70 | 0.00 |
| LIHC | 0.71 | 0.50 | 1.02 | 0.06 | 1.44 | 0.99 | 2.10 | 0.06 | 2.02 | 1.41 | 2.88 | 0.00 | 1.30 | 0.92 | 1.85 | 0.14 | 0.49 | 0.34 | 0.70 | 0.00 | 1.59 | 1.11 | 2.26 | 0.01 | 0.81 | 0.57 | 1.16 | 0.26 |
| LUAD | 0.59 | 0.40 | 0.85 | 0.01 | 1.60 | 1.17 | 2.18 | 0.00 | 1.55 | 1.14 | 2.11 | 0.01 | 1.59 | 1.18 | 2.14 | 0.00 | 0.80 | 0.59 | 1.07 | 0.13 | 0.84 | 0.61 | 1.15 | 0.27 | 1.45 | 1.06 | 1.98 | 0.02 |
| LUSC | 1.47 | 1.10 | 1.97 | 0.01 | 0.72 | 0.55 | 0.96 | 0.02 | 1.21 | 0.92 | 1.60 | 0.18 | 1.27 | 0.93 | 1.74 | 0.14 | 1.36 | 1.01 | 1.83 | 0.05 | 1.45 | 1.11 | 1.91 | 0.01 | 1.15 | 0.88 | 1.51 | 0.31 |
| OV | 0.77 | 0.58 | 1.02 | 0.06 | 1.14 | 0.88 | 1.47 | 0.33 | 0.77 | 0.58 | 1.03 | 0.08 | 1.20 | 0.92 | 1.56 | 0.17 | 1.53 | 1.15 | 2.03 | 0.00 | 1.58 | 1.19 | 2.10 | 0.00 | 1.28 | 0.97 | 1.68 | 0.08 |
| PAAD | 1.80 | 1.18 | 2.75 | 0.01 | 1.39 | 0.82 | 2.36 | 0.22 | 2.21 | 1.44 | 3.37 | 0.00 | 1.96 | 1.24 | 3.11 | 0.00 | 1.41 | 0.93 | 2.13 | 0.11 | 0.58 | 0.37 | 0.90 | 0.01 | 1.54 | 0.97 | 2.46 | 0.07 |
| PCPG | 0.00 | 0.00 | inf | 0.07 |  | 0.00 | inf | 0.00 | 0.60 | 0.12 | 2.97 | 0.53 | 4.40 | 0.51 | 38.11 | 0.14 | 3.11 | 0.35 | 27.72 | 0.29 | 2.65 | 0.53 | 13.32 | 0.22 | 4.26 | 0.50 | 36.50 | 0.15 |
| READ | 0.63 | 0.27 | 1.45 | 0.27 | 1.97 | 0.68 | 5.72 | 0.21 | 0.60 | 0.26 | 1.39 | 0.23 | 1.94 | 0.88 | 4.28 | 0.10 | 1.98 | 0.91 | 4.32 | 0.08 | 2.01 | 0.92 | 4.39 | 0.08 | 0.20 | 0.06 | 0.68 | 0.00 |
| SARC | 0.79 | 0.52 | 1.19 | 0.25 | 1.49 | 0.93 | 2.38 | 0.09 | 1.37 | 0.84 | 2.23 | 0.21 | 0.72 | 0.46 | 1.12 | 0.15 | 0.79 | 0.53 | 1.17 | 0.23 | 0.76 | 0.49 | 1.17 | 0.21 | 1.46 | 0.98 | 2.18 | 0.06 |
| STAD | 1.19 | 0.86 | 1.66 | 0.29 | 1.36 | 0.90 | 2.06 | 0.14 | 1.57 | 1.11 | 2.24 | 0.01 | 0.74 | 0.52 | 1.04 | 0.08 | 0.82 | 0.56 | 1.19 | 0.30 | 1.23 | 0.83 | 1.83 | 0.29 | 0.74 | 0.52 | 1.03 | 0.07 |
| TGCT | 6.44 | 0.66 | 62.52 | 0.07 |  | 0.00 | inf | 0.18 | 2.71 | 0.28 | 26.09 | 0.37 | 0.19 | 0.02 | 1.89 | 0.12 | 0.00 | 0.00 | inf | 0.07 | 4.27 | 0.44 | 41.33 | 0.17 |  | 0.00 | inf | 0.09 |
| THCA | 0.31 | 0.08 | 1.21 | 0.08 | 0.58 | 0.15 | 2.15 | 0.41 | 0.20 | 0.05 | 0.76 | 0.01 | 6.42 | 1.33 | 30.97 | 0.01 | 4.31 | 0.53 | 34.83 | 0.14 | 0.31 | 0.04 | 2.44 | 0.24 | 4.60 | 1.22 | 17.36 | 0.01 |
| THYM | 2.53 | 0.92 | 6.98 | 0.06 | 3.29 | 1.23 | 8.80 | 0.01 | 0.30 | 0.10 | 0.92 | 0.03 | 4.07 | 1.49 | 11.10 | 0.00 | 3.12 | 1.08 | 9.03 | 0.03 | 0.41 | 0.14 | 1.17 | 0.09 | 2.88 | 1.07 | 7.76 | 0.03 |
| UCEC | 1.72 | 1.05 | 2.80 | 0.03 | 1.27 | 0.83 | 1.94 | 0.27 | 1.72 | 1.12 | 2.65 | 0.01 | 0.82 | 0.53 | 1.27 | 0.34 | 1.57 | 1.02 | 2.44 | 0.04 | 1.39 | 0.92 | 2.09 | 0.12 | 1.72 | 1.14 | 2.61 | 0.01 |

Table S3. The HR and 95% confidence interval of the RFS of the SCL39A gene family in pan-cancer derived from KM-plotter dataset.

|  | SCL39A1 | | | | SCL39A2 | | | | SCL39A3 | | | | SCL39A4 | | | | SCL39A5 | | | | SCL39A6 | | | | SCL39A7 | | | |
| --- | --- | --- | --- | --- | --- | --- | --- | --- | --- | --- | --- | --- | --- | --- | --- | --- | --- | --- | --- | --- | --- | --- | --- | --- | --- | --- | --- | --- |
|  | HR | Low | High | P-value | HR | Low | High | P-value | HR | Low | High | P-value | HR | Low | High | P-value | HR | Low | High | P-value | HR | Low | High | P-value | HR | Low | High | P-value |
| BLCA | 1.96 | 0.84 | 4.57 | 0.11 | 0.67 | 0.32 | 1.40 | 0.28 | 0.42 | 0.20 | 0.87 | 0.02 | 0.50 | 0.20 | 1.22 | 0.12 | 0.65 | 0.31 | 1.36 | 0.25 | 1.62 | 0.66 | 3.95 | 0.29 | 0.57 | 0.26 | 1.24 | 0.15 |
| BRCA | 0.68 | 0.44 | 1.04 | 0.08 | 1.32 | 0.85 | 2.05 | 0.21 | 1.46 | 0.93 | 2.28 | 0.10 | 1.46 | 0.95 | 2.26 | 0.08 | 0.67 | 0.43 | 1.05 | 0.08 | 0.45 | 0.29 | 0.69 | 0.00 | 1.46 | 0.94 | 2.25 | 0.09 |
| CESC | 3.41 | 1.28 | 9.07 | 0.01 | 0.34 | 0.16 | 0.74 | 0.00 | 0.47 | 0.21 | 1.09 | 0.07 | 3.01 | 1.13 | 7.98 | 0.02 | 1.65 | 0.76 | 3.57 | 0.20 | 2.41 | 0.83 | 7.03 | 0.10 | 2.58 | 1.19 | 5.61 | 0.01 |
| ESCA | 0.48 | 0.18 | 1.25 | 0.12 | 2.18 | 0.83 | 5.74 | 0.10 | 2.09 | 0.80 | 5.43 | 0.12 | 1.74 | 0.67 | 4.53 | 0.25 | 1.76 | 0.67 | 4.64 | 0.25 | 0.30 | 0.11 | 0.86 | 0.02 | 0.43 | 0.16 | 1.14 | 0.08 |
| HNSC | 2.56 | 1.03 | 6.35 | 0.04 | 1.96 | 0.93 | 4.15 | 0.07 | 1.68 | 0.77 | 3.65 | 0.19 | 0.46 | 0.20 | 1.09 | 0.07 | 0.51 | 0.24 | 1.08 | 0.07 | 0.49 | 0.20 | 1.21 | 0.11 | 0.63 | 0.26 | 1.58 | 0.32 |
| KIRC | 2.16 | 0.48 | 9.66 | 0.30 | 3.89 | 1.09 | 13.86 | 0.02 | 0.49 | 0.18 | 1.35 | 0.16 | 0.40 | 0.13 | 1.16 | 0.08 | 1.79 | 0.50 | 6.36 | 0.36 | 2.67 | 0.95 | 7.52 | 0.05 | 0.44 | 0.15 | 1.26 | 0.12 |
| KIRP | 1.50 | 0.71 | 3.16 | 0.28 | 5.04 | 2.36 | 10.78 | 0.00 | 0.60 | 0.27 | 1.33 | 0.20 | 0.60 | 0.27 | 1.36 | 0.22 | 0.47 | 0.19 | 1.16 | 0.09 | 2.36 | 0.99 | 5.63 | 0.05 | 3.24 | 1.53 | 6.84 | 0.00 |
| LIHC | 1.61 | 1.13 | 2.31 | 0.01 | 0.61 | 0.43 | 0.86 | 0.00 | 0.77 | 0.55 | 1.07 | 0.11 | 0.81 | 0.59 | 1.13 | 0.22 | 0.71 | 0.49 | 1.03 | 0.07 | 1.48 | 1.04 | 2.11 | 0.03 | 1.50 | 1.01 | 2.22 | 0.04 |
| LUAD | 1.58 | 0.94 | 2.66 | 0.08 | 1.42 | 0.91 | 2.21 | 0.12 | 1.37 | 0.89 | 2.09 | 0.15 | 1.43 | 0.91 | 2.24 | 0.12 | 1.32 | 0.87 | 2.02 | 0.19 | 0.78 | 0.51 | 1.19 | 0.25 | 1.21 | 0.79 | 1.86 | 0.37 |
| LUSC | 2.01 | 1.12 | 3.61 | 0.02 | 0.49 | 0.29 | 0.81 | 0.00 | 0.67 | 0.39 | 1.13 | 0.13 | 1.56 | 0.93 | 2.60 | 0.09 | 0.52 | 0.26 | 1.07 | 0.07 | 1.63 | 0.97 | 2.73 | 0.06 | 1.34 | 0.81 | 2.23 | 0.25 |
| OV | 0.83 | 0.58 | 1.19 | 0.30 | 0.69 | 0.46 | 1.06 | 0.09 | 0.48 | 0.33 | 0.70 | 0.00 | 0.83 | 0.55 | 1.24 | 0.36 | 0.73 | 0.51 | 1.05 | 0.09 | 1.19 | 0.84 | 1.69 | 0.33 | 0.67 | 0.45 | 1.00 | 0.05 |
| PAAD | 3.52 | 1.30 | 9.53 | 0.01 | 4.66 | 1.37 | 15.92 | 0.01 | 0.40 | 0.16 | 1.01 | 0.05 | 2.78 | 1.22 | 6.33 | 0.01 | 1.79 | 0.70 | 4.61 | 0.22 | 1.92 | 0.57 | 6.48 | 0.28 | 0.43 | 0.16 | 1.17 | 0.09 |
| PCPG | 3.68 | 0.45 | 30.17 | 0.20 |  | 0.00 | inf | 0.06 | 0.12 | 0.01 | 1.18 | 0.03 | 0.12 | 0.01 | 1.16 | 0.03 | 0.50 | 0.07 | 3.56 | 0.48 | 2.68 | 0.38 | 19.05 | 0.31 | 8.78 | 0.89 | 86.15 | 0.03 |
| READ | 3.75 | 0.73 | 19.22 | 0.09 | 0.20 | 0.03 | 1.52 | 0.09 | 0.42 | 0.05 | 3.67 | 0.42 | 0.35 | 0.07 | 1.79 | 0.19 | 0.40 | 0.08 | 2.04 | 0.25 | 4.16 | 0.75 | 22.94 | 0.08 | 2.79 | 0.32 | 24.06 | 0.33 |
| SARC | 0.52 | 0.31 | 0.87 | 0.01 | 0.53 | 0.31 | 0.89 | 0.02 | 0.55 | 0.30 | 1.00 | 0.05 | 1.17 | 0.68 | 2.02 | 0.56 | 0.50 | 0.27 | 0.90 | 0.02 | 1.42 | 0.88 | 2.31 | 0.15 | 0.70 | 0.43 | 1.15 | 0.16 |
| STAD | 1.62 | 0.71 | 3.70 | 0.24 | 2.05 | 1.04 | 4.04 | 0.03 | 0.42 | 0.16 | 1.07 | 0.06 | 2.10 | 1.07 | 4.11 | 0.03 | 0.62 | 0.32 | 1.18 | 0.14 | 0.58 | 0.30 | 1.11 | 0.10 | 1.81 | 0.89 | 3.66 | 0.10 |
| TGCT | 2.06 | 0.96 | 4.42 | 0.06 | 2.59 | 1.21 | 5.56 | 0.01 | 1.34 | 0.59 | 3.07 | 0.49 | 1.93 | 0.90 | 4.17 | 0.09 | 2.56 | 1.19 | 5.47 | 0.01 | 0.47 | 0.22 | 1.02 | 0.05 | 2.82 | 0.85 | 9.38 | 0.08 |
| THCA | 0.29 | 0.13 | 0.66 | 0.00 | 2.23 | 1.03 | 4.86 | 0.04 | 0.45 | 0.16 | 1.31 | 0.13 | 0.69 | 0.32 | 1.48 | 0.34 | 0.60 | 0.28 | 1.31 | 0.20 | 1.82 | 0.83 | 4.01 | 0.13 | 0.36 | 0.17 | 0.77 | 0.01 |
| UCEC | 1.40 | 0.83 | 2.35 | 0.20 | 0.68 | 0.40 | 1.14 | 0.14 | 0.61 | 0.35 | 1.06 | 0.08 | 1.99 | 1.03 | 3.84 | 0.04 | 1.69 | 0.97 | 2.93 | 0.06 | 0.74 | 0.39 | 1.40 | 0.36 | 0.61 | 0.36 | 1.03 | 0.06 |
|  | SCL39A8 | | | | SCL39A9 | | | | SCL39A10 | | | | SCL39A11 | | | | SCL39A12 | | | | SCL39A13 | | | | SCL39A14 | | | |
|  | HR | Low | High | P-value | HR | Low | High | P-value | HR | Low | High | P-value | HR | Low | High | P-value | HR | Low | High | P-value | HR | Low | High | P-value | HR | Low | High | P-value |
| BLCA | 2.33 | 0.82 | 6.67 | 0.10 | 1.87 | 0.90 | 3.86 | 0.09 | 1.75 | 0.80 | 3.84 | 0.15 | 1.62 | 0.76 | 3.44 | 0.21 | 1.55 | 0.77 | 3.14 | 0.22 | 0.54 | 0.26 | 1.11 | 0.09 | 1.60 | 0.78 | 3.28 | 0.19 |
| BRCA | 0.55 | 0.35 | 0.86 | 0.01 | 0.84 | 0.54 | 1.30 | 0.43 | 0.67 | 0.43 | 1.03 | 0.07 | 0.68 | 0.41 | 1.11 | 0.12 | 1.38 | 0.90 | 2.12 | 0.13 | 0.78 | 0.51 | 1.21 | 0.27 | 1.33 | 0.84 | 2.12 | 0.22 |
| CESC | 1.78 | 0.79 | 4.00 | 0.16 | 3.02 | 1.39 | 6.56 | 0.00 | 3.97 | 1.83 | 8.62 | 0.00 | 2.23 | 0.77 | 6.49 | 0.13 | 2.03 | 0.94 | 4.40 | 0.07 | 2.31 | 1.00 | 5.34 | 0.04 | 2.70 | 0.93 | 7.84 | 0.06 |
| ESCA | 0.48 | 0.14 | 1.67 | 0.24 | 2.68 | 0.61 | 11.73 | 0.17 | 1.46 | 0.54 | 3.96 | 0.45 | 0.50 | 0.19 | 1.31 | 0.15 | 0.16 | 0.02 | 1.18 | 0.04 | 2.08 | 0.80 | 5.41 | 0.13 | 0.41 | 0.16 | 1.06 | 0.06 |
| HNSC | 1.81 | 0.85 | 3.82 | 0.12 | 0.54 | 0.21 | 1.43 | 0.21 | 2.36 | 0.82 | 6.84 | 0.10 | 0.44 | 0.15 | 1.26 | 0.11 | 0.23 | 0.06 | 0.98 | 0.03 | 1.84 | 0.87 | 3.90 | 0.10 | 3.67 | 1.68 | 8.02 | 0.00 |
| KIRC | 0.37 | 0.14 | 1.03 | 0.05 | 0.33 | 0.09 | 1.17 | 0.07 | 0.64 | 0.23 | 1.80 | 0.39 | 7.75 | 1.75 | 34.34 | 0.00 | 0.39 | 0.11 | 1.40 | 0.14 | 2.66 | 0.60 | 11.81 | 0.18 | 2.22 | 0.80 | 6.14 | 0.12 |
| KIRP | 0.60 | 0.27 | 1.33 | 0.20 | 1.89 | 0.88 | 4.06 | 0.10 | 2.48 | 1.08 | 5.70 | 0.03 | 1.84 | 0.80 | 4.23 | 0.15 | 2.10 | 1.00 | 4.43 | 0.05 | 0.38 | 0.18 | 0.83 | 0.01 | 3.47 | 1.20 | 10.02 | 0.01 |
| LIHC | 1.28 | 0.91 | 1.79 | 0.15 | 0.58 | 0.41 | 0.83 | 0.00 | 1.50 | 1.04 | 2.15 | 0.03 | 0.76 | 0.54 | 1.05 | 0.10 | 0.65 | 0.46 | 0.91 | 0.01 | 1.44 | 1.03 | 2.00 | 0.03 | 0.77 | 0.53 | 1.10 | 0.15 |
| LUAD | 0.72 | 0.43 | 1.20 | 0.21 | 1.50 | 0.97 | 2.32 | 0.07 | 1.51 | 0.97 | 2.34 | 0.06 | 2.05 | 1.33 | 3.17 | 0.00 | 1.82 | 1.18 | 2.79 | 0.01 | 1.66 | 0.98 | 2.82 | 0.06 | 1.36 | 0.86 | 2.15 | 0.19 |
| LUSC | 1.29 | 0.78 | 2.14 | 0.32 | 0.61 | 0.37 | 1.02 | 0.06 | 1.56 | 0.94 | 2.61 | 0.08 | 1.61 | 0.87 | 2.98 | 0.12 | 1.56 | 0.93 | 2.62 | 0.09 | 1.25 | 0.72 | 2.17 | 0.42 | 0.72 | 0.43 | 1.19 | 0.19 |
| OV | 0.75 | 0.52 | 1.07 | 0.11 | 0.71 | 0.50 | 1.01 | 0.05 | 0.88 | 0.61 | 1.26 | 0.48 | 0.66 | 0.45 | 0.95 | 0.03 | 0.61 | 0.40 | 0.92 | 0.02 | 1.27 | 0.85 | 1.91 | 0.24 | 1.55 | 1.08 | 2.23 | 0.02 |
| PAAD | 4.27 | 1.36 | 13.42 | 0.01 | 1.99 | 0.87 | 4.52 | 0.10 | 11.13 | 2.53 | 48.92 | 0.00 | 12.54 | 1.69 | 93.10 | 0.00 | 0.65 | 0.28 | 1.53 | 0.32 | 2.02 | 0.89 | 4.59 | 0.09 | 15.07 | 1.91 | 119.23 | 0.00 |
| PCPG | 4.08 | 0.42 | 39.61 | 0.19 | 9.87 | 1.01 | 96.04 | 0.02 |  | 0.00 | inf | 0.09 | 2.53 | 0.35 | 18.57 | 0.34 | 0.44 | 0.06 | 3.20 | 0.41 | 0.26 | 0.03 | 2.49 | 0.21 | 6.08 | 0.59 | 62.82 | 0.09 |
| READ | 0.16 | 0.02 | 1.40 | 0.06 | 2.76 | 0.32 | 23.98 | 0.34 |  | 0.00 | inf | 0.11 | 0.20 | 0.02 | 1.73 | 0.11 | 2.91 | 0.34 | 25.19 | 0.31 | 3.11 | 0.56 | 17.19 | 0.17 | 3.62 | 0.42 | 31.03 | 0.21 |
| SARC | 1.23 | 0.74 | 2.03 | 0.42 | 1.43 | 0.87 | 2.35 | 0.15 | 0.51 | 0.28 | 0.92 | 0.02 | 0.68 | 0.42 | 1.11 | 0.12 | 0.78 | 0.48 | 1.27 | 0.32 | 1.23 | 0.75 | 2.03 | 0.41 | 0.67 | 0.37 | 1.22 | 0.19 |
| STAD | 0.28 | 0.12 | 0.68 | 0.00 | 0.51 | 0.25 | 1.05 | 0.06 | 0.43 | 0.18 | 1.03 | 0.05 | 0.78 | 0.40 | 1.54 | 0.47 | 2.29 | 1.18 | 4.46 | 0.01 | 1.98 | 0.90 | 4.33 | 0.08 | 1.50 | 0.77 | 2.92 | 0.23 |
| TGCT | 1.86 | 0.70 | 4.93 | 0.21 | 1.92 | 0.90 | 4.10 | 0.09 | 2.86 | 0.86 | 9.50 | 0.07 | 0.50 | 0.21 | 1.18 | 0.11 | 0.54 | 0.25 | 1.16 | 0.11 | 1.72 | 0.75 | 3.93 | 0.19 | 2.79 | 1.12 | 6.91 | 0.02 |
| THCA | 2.18 | 1.00 | 4.74 | 0.05 | 0.55 | 0.19 | 1.59 | 0.26 | 0.45 | 0.16 | 1.32 | 0.14 | 2.48 | 0.85 | 7.19 | 0.09 | 1.77 | 0.80 | 3.90 | 0.15 | 2.93 | 1.11 | 7.78 | 23.00 | 0.52 | 0.19 | 1.37 | 0.18 |
| UCEC | 0.55 | 0.33 | 0.93 | 0.02 | 1.53 | 0.91 | 2.59 | 0.11 | 2.09 | 1.22 | 3.56 | 0.01 | 0.76 | 0.44 | 1.31 | 0.32 | 0.35 | 0.17 | 0.72 | 0.00 | 1.39 | 0.75 | 2.58 | 0.30 | 2.20 | 1.22 | 3.96 | 0.01 |

**Table S4. Survival analysis of the prognostic value of SLC39A family genes in breast, colorectal, lung and ovarian cancer derived from PrognoScan dataset.**

| ID_NAME | DATASET | CANCER TYPE | ENDPOINT | N | COX P-VALUE | HR | 95% CI |
| --- | --- | --- | --- | --- | --- | --- | --- |
| SLC39A1 | GSE1456-GPL96 | Breast cancer | DSS | 159 | 0.003 | 7.21 | [2.00 - 25.99] |
| SLC39A1 | GSE1456-GPL96 | Breast cancer | OS | 159 | 0.006 | 4.52 | [1.53 - 13.37] |
| SLC39A11 | GSE1456-GPL97 | Breast cancer | DSS | 159 | 0.007 | 2.72 | [1.32 - 5.60] |
| SLC39A11 | GSE1456-GPL97 | Breast cancer | OS | 159 | 0.045 | 1.91 | [1.01 - 3.60] |
| SLC39A11 | GSE1456-GPL97 | Breast cancer | RFS | 159 | 0.007 | 2.22 | [1.24 - 3.96] |
| SLC39A14 | GSE7849 | Breast cancer | DFS | 76 | 0.031 | 6.64 | [1.19 - 36.87] |
| SLC39A14 | GSE9195 | Breast cancer | DMFS | 77 | 0.042 | 0.02 | [0.00 - 0.87] |
| SLC39A14 | GSE1378 | Breast cancer | RFS | 60 | 0.044 | 3.84 | [1.04 - 14.23] |
| SLC39A2 | GSE1456-GPL96 | Breast cancer | DSS | 159 | 0.048 | 1.64 | [1.00 - 2.66] |
| SLC39A2 | GSE1456-GPL96 | Breast cancer | OS | 159 | 0.044 | 1.54 | [1.01 - 2.34] |
| SLC39A2 | GSE1456-GPL96 | Breast cancer | RFS | 159 | 0.012 | 1.69 | [1.12 - 2.55] |
| SLC39A3 | GSE4922-GPL97 | Breast cancer | DFS | 249 | 0.028 | 1.41 | [1.04 - 1.90] |
| SLC39A3 | GSE3494-GPL97 | Breast cancer | DSS | 236 | 0.039 | 1.5 | [1.02 - 2.19] |
| SLC39A3 | GSE9195 | Breast cancer | RFS | 77 | 0.025 | 13.31 | [1.39 - 127.14] |
| SLC39A3 | GSE1456-GPL97 | Breast cancer | RFS | 159 | 0.041 | 1.57 | [1.02 - 2.43] |
| SLC39A3 | GSE1379 | Breast cancer | RFS | 60 | 0.045 | 2.67 | [1.02 - 6.98] |
| SLC39A4 | GSE1456-GPL96 | Breast cancer | DSS | 159 | 0.030 | 1.63 | [1.05 - 2.54] |
| SLC39A4 | GSE2034 | Breast cancer | DMFS | 286 | 0.000 | 1.59 | [1.24 - 2.04] |
| SLC39A4 | GSE11121 | Breast cancer | DMFS | 200 | 0.030 | 1.61 | [1.05 - 2.47] |
| SLC39A4 | GSE12093 | Breast cancer | DMFS | 136 | 0.042 | 1.79 | [1.02 - 3.15] |
| SLC39A4 | GSE1456-GPL96 | Breast cancer | RFS | 159 | 0.005 | 1.72 | [1.18 - 2.50] |
| SLC39A5 | GSE1456-GPL97 | Breast cancer | DSS | 159 | 0.026 | 2.69 | [1.12 - 6.43] |
| SLC39A5 | GSE1456-GPL97 | Breast cancer | OS | 159 | 0.046 | 2.15 | [1.01 - 4.56] |
| SLC39A5 | GSE1456-GPL97 | Breast cancer | RFS | 159 | 0.018 | 2.48 | [1.17 - 5.26] |
| SLC39A6 | GSE11121 | Breast cancer | DMFS | 200 | 0.004 | 0.62 | [0.45 - 0.86] |
| SLC39A6 | GSE11121 | Breast cancer | DMFS | 200 | 0.012 | 0.59 | [0.39 - 0.89] |
| SLC39A6 | GSE19615 | Breast cancer | DMFS | 115 | 0.020 | 0.42 | [0.20 - 0.87] |
| SLC39A6 | GSE12276 | Breast cancer | RFS | 204 | 0.000 | 0.7 | [0.58 - 0.85] |
| SLC39A6 | GSE12276 | Breast cancer | RFS | 204 | 0.001 | 0.79 | [0.69 - 0.91] |
| SLC39A6 | GSE12276 | Breast cancer | RFS | 204 | 0.002 | 0.83 | [0.74 - 0.93] |
| SLC39A6 | GSE12276 | Breast cancer | RFS | 204 | 0.004 | 0.85 | [0.76 - 0.95] |
| SLC39A7 | GSE4922-GPL96 | Breast cancer | DFS | 249 | 0.001 | 3.11 | [1.57 - 6.17] |
| SLC39A7 | GSE3494-GPL96 | Breast cancer | DSS | 236 | 0.001 | 4.93 | [1.99 - 12.17] |
| SLC39A7 | GSE11121 | Breast cancer | DMFS | 200 | 0.005 | 3.96 | [1.51 - 10.38] |
| SLC39A7 | GSE12276 | Breast cancer | RFS | 204 | 0.015 | 1.5 | [1.08 - 2.08] |
| SLC39A8 | GSE7390 | Breast cancer | DMFS | 198 | 0.031 | 1.37 | [1.03 - 1.82] |
| SLC39A8 | GSE11121 | Breast cancer | DMFS | 200 | 0.031 | 1.51 | [1.04 - 2.21] |
| SLC39A8 | GSE7390 | Breast cancer | OS | 198 | 0.044 | 1.36 | [1.01 - 1.84] |
| SLC39A8 | GSE12276 | Breast cancer | RFS | 204 | 0.005 | 1.28 | [1.08 - 1.53] |
| SLC39A8 | GSE12276 | Breast cancer | RFS | 204 | 0.012 | 1.23 | [1.05 - 1.45] |
| SLC39A8 | GSE7390 | Breast cancer | RFS | 198 | 0.017 | 1.32 | [1.05 - 1.66] |
| SLC39A8 | GSE1456-GPL96 | Breast cancer | RFS | 159 | 0.044 | 1.71 | [1.01 - 2.90] |
| SLC39A9 | GSE7390 | Breast cancer | DMFS | 198 | 0.025 | 1.63 | [1.06 - 2.51] |
| SLC39A9 | GSE7390 | Breast cancer | OS | 198 | 0.043 | 1.57 | [1.02 - 2.43] |
| SLC39A9 | GSE7390 | Breast cancer | RFS | 198 | 0.009 | 1.6 | [1.12 - 2.27] |
| SLC39A1 | GSE17537 | Colorectal cancer | DFS | 55 | 0.030 | 11.24 | [1.27 - 99.27] |
| SLC39A10 | GSE17537 | Colorectal cancer | DFS | 55 | 0.039 | 3 | [1.06 - 8.51] |
| SLC39A10 | GSE17537 | Colorectal cancer | DSS | 49 | 0.018 | 5.46 | [1.33 - 22.36] |
| SLC39A13 | GSE14333 | Colorectal cancer | DFS | 226 | 0.046 | 1.89 | [1.01 - 3.52] |
| SLC39A13 | GSE17537 | Colorectal cancer | DSS | 49 | 0.045 | 0.11 | [0.01 - 0.95] |
| SLC39A14 | GSE12945 | Colorectal cancer | DFS | 51 | 0.002 | 0.19 | [0.07 - 0.55] |
| SLC39A14 | GSE17537 | Colorectal cancer | DSS | 49 | 0.038 | 0.42 | [0.19 - 0.95] |
| SLC39A3 | GSE17537 | Colorectal cancer | DFS | 55 | 0.001 | 0.01 | [0.00 - 0.16] |
| SLC39A3 | GSE17537 | Colorectal cancer | DSS | 49 | 0.019 | 0.01 | [0.00 - 0.44] |
| SLC39A3 | GSE17537 | Colorectal cancer | OS | 55 | 0.014 | 0.03 | [0.00 - 0.50] |
| SLC39A5 | GSE14333 | Colorectal cancer | DFS | 226 | 0.004 | 0.7 | [0.55 - 0.90] |
| SLC39A5 | GSE17536 | Colorectal cancer | DFS | 145 | 0.006 | 0.31 | [0.14 - 0.71] |
| SLC39A5 | GSE17536 | Colorectal cancer | DFS | 145 | 0.019 | 0.36 | [0.16 - 0.84] |
| SLC39A5 | GSE14333 | Colorectal cancer | DFS | 226 | 0.026 | 0.81 | [0.67 - 0.97] |
| SLC39A5 | GSE17536 | Colorectal cancer | DSS | 177 | 0.005 | 0.39 | [0.20 - 0.75] |
| SLC39A5 | GSE17536 | Colorectal cancer | DSS | 177 | 0.007 | 0.38 | [0.18 - 0.77] |
| SLC39A5 | GSE17536 | Colorectal cancer | OS | 177 | 0.018 | 0.5 | [0.29 - 0.89] |
| SLC39A5 | GSE17536 | Colorectal cancer | OS | 177 | 0.033 | 0.51 | [0.27 - 0.95] |
| SLC39A6 | GSE14333 | Colorectal cancer | DFS | 226 | 0.026 | 1.71 | [1.07 - 2.75] |
| SLC39A6 | GSE17536 | Colorectal cancer | DFS | 145 | 0.030 | 1.99 | [1.07 - 3.69] |
| SLC39A6 | GSE17536 | Colorectal cancer | DFS | 145 | 0.047 | 2.04 | [1.01 - 4.11] |
| SLC39A6 | GSE17536 | Colorectal cancer | DFS | 145 | 0.049 | 2.35 | [1.00 - 5.49] |
| SLC39A6 | GSE17536 | Colorectal cancer | DSS | 177 | 0.049 | 1.63 | [1.00 - 2.66] |
| SLC39A6 | GSE17537 | Colorectal cancer | OS | 55 | 0.046 | 2.09 | [1.01 - 4.31] |
| SLC39A6 | GSE17536 | Colorectal cancer | OS | 177 | 0.050 | 1.61 | [1.00 - 2.59] |
| SLC39A7 | GSE17537 | Colorectal cancer | DFS | 55 | 0.005 | 0.27 | [0.11 - 0.68] |
| SLC39A8 | GSE14333 | Colorectal cancer | DFS | 226 | 0.000 | 0.52 | [0.38 - 0.71] |
| SLC39A8 | GSE14333 | Colorectal cancer | DFS | 226 | 0.003 | 0.63 | [0.47 - 0.86] |
| SLC39A8 | GSE14333 | Colorectal cancer | DFS | 226 | 0.004 | 0.7 | [0.54 - 0.89] |
| SLC39A8 | GSE14333 | Colorectal cancer | DFS | 226 | 0.010 | 0.78 | [0.65 - 0.94] |
| SLC39A8 | GSE17536 | Colorectal cancer | DFS | 145 | 0.015 | 0.62 | [0.42 - 0.91] |
| SLC39A8 | GSE14333 | Colorectal cancer | DFS | 226 | 0.015 | 0.68 | [0.50 - 0.93] |
| SLC39A8 | GSE17537 | Colorectal cancer | DFS | 55 | 0.021 | 0.53 | [0.31 - 0.91] |
| SLC39A8 | GSE17537 | Colorectal cancer | DFS | 55 | 0.031 | 0.57 | [0.34 - 0.95] |
| SLC39A8 | GSE17536 | Colorectal cancer | DFS | 145 | 0.043 | 0.19 | [0.04 - 0.95] |
| SLC39A8 | GSE17536 | Colorectal cancer | DFS | 145 | 0.044 | 0.66 | [0.44 - 0.99] |
| SLC39A8 | GSE17537 | Colorectal cancer | DFS | 55 | 0.045 | 0.18 | [0.04 - 0.96] |
| SLC39A8 | GSE17536 | Colorectal cancer | DFS | 145 | 0.050 | 0.19 | [0.03 - 1.00] |
| SLC39A8 | GSE17536 | Colorectal cancer | DSS | 177 | 0.001 | 0.1 | [0.03 - 0.38] |
| SLC39A8 | GSE17536 | Colorectal cancer | DSS | 177 | 0.004 | 0.65 | [0.48 - 0.87] |
| SLC39A8 | GSE17536 | Colorectal cancer | DSS | 177 | 0.004 | 0.62 | [0.45 - 0.86] |
| SLC39A8 | GSE17536 | Colorectal cancer | DSS | 177 | 0.008 | 0.16 | [0.04 - 0.62] |
| SLC39A8 | GSE17536 | Colorectal cancer | DSS | 177 | 0.019 | 0.22 | [0.06 - 0.78] |
| SLC39A8 | GSE17537 | Colorectal cancer | DSS | 49 | 0.038 | 0.5 | [0.26 - 0.96] |
| SLC39A8 | GSE17536 | Colorectal cancer | DSS | 177 | 0.045 | 0.56 | [0.31 - 0.99] |
| SLC39A8 | GSE17536 | Colorectal cancer | OS | 177 | 0.000 | 0.12 | [0.04 - 0.37] |
| SLC39A8 | GSE17536 | Colorectal cancer | OS | 177 | 0.006 | 0.67 | [0.51 - 0.89] |
| SLC39A8 | GSE17536 | Colorectal cancer | OS | 177 | 0.006 | 0.19 | [0.06 - 0.63] |
| SLC39A8 | GSE17537 | Colorectal cancer | OS | 55 | 0.014 | 0.54 | [0.34 - 0.88] |
| SLC39A8 | GSE17536 | Colorectal cancer | OS | 177 | 0.014 | 0.72 | [0.55 - 0.93] |
| SLC39A8 | GSE12945 | Colorectal cancer | OS | 62 | 0.019 | 0.26 | [0.08 - 0.80] |
| SLC39A8 | GSE17536 | Colorectal cancer | OS | 177 | 0.022 | 0.28 | [0.09 - 0.83] |
| SLC39A8 | GSE17537 | Colorectal cancer | OS | 55 | 0.031 | 0.19 | [0.04 - 0.85] |
| SLC39A8 | GSE17537 | Colorectal cancer | OS | 55 | 0.032 | 0.57 | [0.34 - 0.95] |
| SLC39A9 | GSE17536 | Colorectal cancer | DSS | 177 | 0.000 | 5.77 | [2.18 - 15.28] |
| SLC39A9 | GSE17536 | Colorectal cancer | OS | 177 | 0.001 | 3.98 | [1.73 - 9.15] |
| SLC39A1 | GSE31210 | Lung cancer | OS | 204 | 0.000 | 9.59 | [2.71 - 33.97] |
| SLC39A1 | jacob-00182-MSK | Lung cancer | OS | 104 | 0.004 | 7.3 | [1.85 - 28.71] |
| SLC39A1 | GSE31210 | Lung cancer | RFS | 204 | 0.000 | 9.15 | [3.43 - 24.40] |
| SLC39A1 | GSE8894 | Lung cancer | RFS | 138 | 0.039 | 1.75 | [1.03 - 2.97] |
| SLC39A10 | GSE13213 | Lung cancer | OS | 117 | 0.002 | 1.78 | [1.24 - 2.56] |
| SLC39A10 | GSE8894 | Lung cancer | RFS | 138 | 0.046 | 1.4 | [1.01 - 1.94] |
| SLC39A11 | GSE31210 | Lung cancer | OS | 204 | 0.001 | 4.3 | [1.78 - 10.40] |
| SLC39A11 | GSE31210 | Lung cancer | RFS | 204 | 0.000 | 4.59 | [2.35 - 8.96] |
| SLC39A12 | GSE31210 | Lung cancer | RFS | 204 | 0.037 | 0.68 | [0.48 - 0.98] |
| SLC39A13 | GSE31210 | Lung cancer | RFS | 204 | 0.005 | 3.93 | [1.51 - 10.25] |
| SLC39A14 | GSE31210 | Lung cancer | RFS | 204 | 0.004 | 1.82 | [1.21 - 2.73] |
| SLC39A3 | GSE11117 | Lung cancer | OS | 41 | 0.004 | 3.39 | [1.47 - 7.84] |
| SLC39A3 | GSE13213 | Lung cancer | OS | 117 | 0.035 | 0.51 | [0.27 - 0.95] |
| SLC39A4 | GSE31210 | Lung cancer | OS | 204 | 0.018 | 1.89 | [1.11 - 3.20] |
| SLC39A4 | GSE4573 | Lung cancer | OS | 129 | 0.019 | 1.72 | [1.09 - 2.71] |
| SLC39A4 | GSE31210 | Lung cancer | RFS | 204 | 0.001 | 1.97 | [1.32 - 2.94] |
| SLC39A6 | GSE31210 | Lung cancer | OS | 204 | 0.000 | 3.67 | [1.77 - 7.61] |
| SLC39A6 | GSE31210 | Lung cancer | OS | 204 | 0.002 | 3.18 | [1.51 - 6.70] |
| SLC39A6 | GSE31210 | Lung cancer | OS | 204 | 0.010 | 2.58 | [1.25 - 5.33] |
| SLC39A6 | GSE31210 | Lung cancer | OS | 204 | 0.020 | 3.74 | [1.23 - 11.35] |
| SLC39A6 | GSE31210 | Lung cancer | RFS | 204 | 0.000 | 4.28 | [2.53 - 7.25] |
| SLC39A6 | GSE31210 | Lung cancer | RFS | 204 | 0.000 | 3.52 | [2.02 - 6.13] |
| SLC39A6 | GSE31210 | Lung cancer | RFS | 204 | 0.000 | 5.68 | [2.63 - 12.28] |
| SLC39A6 | GSE31210 | Lung cancer | RFS | 204 | 0.000 | 3.12 | [1.86 - 5.24] |
| SLC39A8 | GSE31210 | Lung cancer | OS | 204 | 0.009 | 0.26 | [0.09 - 0.71] |
| SLC39A8 | GSE3141 | Lung cancer | OS | 111 | 0.032 | 1.43 | [1.03 - 1.98] |
| SLC39A8 | GSE31210 | Lung cancer | RFS | 204 | 0.002 | 0.48 | [0.30 - 0.77] |
| SLC39A8 | GSE31210 | Lung cancer | RFS | 204 | 0.006 | 0.48 | [0.29 - 0.81] |
| SLC39A8 | GSE31210 | Lung cancer | RFS | 204 | 0.013 | 0.44 | [0.23 - 0.84] |
| SLC39A8 | GSE8894 | Lung cancer | RFS | 138 | 0.045 | 1.14 | [1.00 - 1.30] |
| SLC39A1 | DUKE-OC | Ovarian cancer | OS | 133 | 0.034 | 1.65 | [1.04 - 2.63] |
| SLC39A10 | GSE9891 | Ovarian cancer | OS | 278 | 0.032 | 1.34 | [1.02 - 1.75] |
| SLC39A13 | GSE9891 | Ovarian cancer | OS | 278 | 0.000 | 3.21 | [1.83 - 5.64] |
| SLC39A13 | GSE8841 | Ovarian cancer | OS | 81 | 0.015 | 3.62 | [1.28 - 10.20] |
| SLC39A13 | GSE9891 | Ovarian cancer | OS | 278 | 0.021 | 1.66 | [1.08 - 2.55] |
| SLC39A13 | GSE17260 | Ovarian cancer | PFS | 110 | 0.019 | 1.78 | [1.10 - 2.86] |
| SLC39A14 | GSE26712 | Ovarian cancer | DFS | 185 | 0.019 | 1.31 | [1.05 - 1.64] |
| SLC39A14 | GSE26712 | Ovarian cancer | OS | 185 | 0.024 | 1.32 | [1.04 - 1.69] |
| SLC39A14 | GSE9891 | Ovarian cancer | OS | 278 | 0.045 | 1.29 | [1.01 - 1.65] |
| SLC39A4 | GSE26712 | Ovarian cancer | DFS | 185 | 0.036 | 0.85 | [0.72 - 0.99] |
| SLC39A4 | GSE26712 | Ovarian cancer | OS | 185 | 0.021 | 0.82 | [0.69 - 0.97] |
| SLC39A5 | GSE17260 | Ovarian cancer | OS | 110 | 0.021 | 0.5 | [0.28 - 0.90] |
| SLC39A8 | GSE26712 | Ovarian cancer | DFS | 185 | 0.002 | 0.52 | [0.34 - 0.78] |
| SLC39A8 | DUKE-OC | Ovarian cancer | OS | 133 | 0.004 | 1.73 | [1.19 - 2.52] |
| SLC39A8 | GSE26712 | Ovarian cancer | OS | 185 | 0.019 | 0.6 | [0.39 - 0.92] |
| SLC39A9 | GSE14764 | Ovarian cancer | OS | 80 | 0.027 | 2.08 | [1.09 - 3.99] |

OS: overall survival; PFS: progression free survival; RFS: relapse-free survival; DRFS: distant recurrence-free survival; DFS: disease-free survival; DSS: disease-specific survival; DMFS: distant metastasis-free survival.

Table S5. The STRING and GENEMAIN databases were used to identify SLC39A family genes highly related genes.

| **STRING** | **GeneMain** | **Union** | **Intersection** |
| --- | --- | --- | --- |
| AKR1D1 | SCL39A6 | AKR1D1 | SLC39A1 |
| ARFIP1 | SCL39A4 | ARFIP1 | SLC39A10 |
| CBR4 | SCL39A5 | CBR4 | SLC39A11 |
| CYBRD1 | SPINT2 | CYBRD1 | SLC39A12 |
| CYP11B1 | PGAP4 | CYP11B1 | SLC39A13 |
| CYP11B2 | CYSTM1 | CYP11B2 | SLC39A14 |
| CYP17A1 | SCL39A8 | CYP17A1 | SLC39A2 |
| CYP19A1 | SCL39A1 | CYP19A1 | SLC39A3 |
| CYP1A1 | KCNAB1 | CYP1A1 | SLC39A4 |
| CYP1B1 | AGK | CYP1B1 | SLC39A |
| CYP3A4 | APOOL | CYP3A4 |  |
| CYP3A5 | SCL39A9 | CYP3A5 |  |
| HAMP | SCL39A11 | HAMP |  |
| HEPH | KCNAB2 | HEPH |  |
| HSD17B1 | SPP1 | HSD17B1 |  |
| HSD17B12 | VKORC1L1 | HSD17B12 |  |
| HSD17B3 | BSCL2 | HSD17B3 |  |
| HSD17B6 | SYAP1 | HSD17B6 |  |
| HSD17B7 | POM121C | HSD17B7 |  |
| HSD17B8 | SPN | HSD17B8 |  |
| HSD3B1 | SYNGR3 | HSD3B1 |  |
| HSD3B2 | C1GALT1C1 | HSD3B2 |  |
| MCAT | METTL16 | MCAT |  |
| PTPRN | C3orf33 | PTPRN |  |
| SLC11A2 | FAM241B | SLC11A2 |  |
| SLC30A1 | SCL39A14 | SLC30A1 |  |
| SLC30A10 | SCL39A10 | SLC30A10 |  |
| SLC30A2 | SCL39A2 | SLC30A2 |  |
| SLC30A4 | SCL39A3 | SLC30A4 |  |
| SLC30A5 | SCL39A7 | SLC30A5 |  |
| SLC30A6 | SCL39A12 | SLC30A6 |  |
| SLC30A7 | AC006538.2 | SLC30A7 |  |
| SLC30A9 | FAM241A | SLC30A9 |  |
| SCL39A1 | SCL39A13 | SCL39A1 |  |
| SCL39A10 |  | SCL39A10 |  |
| SCL39A11 |  | SCL39A11 |  |
| SCL39A12 |  | SCL39A12 |  |
| SCL39A13 |  | SCL39A13 |  |
| SCL39A14 |  | SCL39A14 |  |
| SCL39A2 |  | SCL39A2 |  |
| SCL39A3 |  | SCL39A3 |  |
| SCL39A4 |  | SCL39A4 |  |
| SCL39A5 |  | SCL39A5 |  |
| SCL39A6 |  | SCL39A6 |  |
| SCL39A7 |  | SCL39A7 |  |
| SCL39A8 |  | SCL39A8 |  |
| SLC40A1 |  | SLC40A1 |  |
| SRD5A1 |  | SRD5A1 |  |
| SRD5A2 |  | SRD5A2 |  |
| SRD5A3 |  | SRD5A3 |  |
| OXSM |  | OXSM |  |
| TFRC |  | TFRC |  |
| SULT1E1 |  | SULT1E1 |  |
| SCL39A9 |  | SCL39A9 |  |
|  |  | SPINT2 |  |
|  |  | PGAP4 |  |
|  |  | CYSTM1 |  |
|  |  | KCNAB1 |  |
|  |  | AGK |  |
|  |  | APOOL |  |
|  |  | KCNAB2 |  |
|  |  | SPP1 |  |
|  |  | VKORC1L1 |  |
|  |  | BSCL2 |  |
|  |  | SYAP1 |  |
|  |  | POM121C |  |
|  |  | SPN |  |
|  |  | SYNGR3 |  |
|  |  | C1GALT1C1 |  |
|  |  | METTL16 |  |
|  |  | C3orf33 |  |
|  |  | FAM241B |  |
|  |  | AC006538.2 |  |
|  |  | FAM241A |  |

Figure S1. The methodical workflow of the current research.


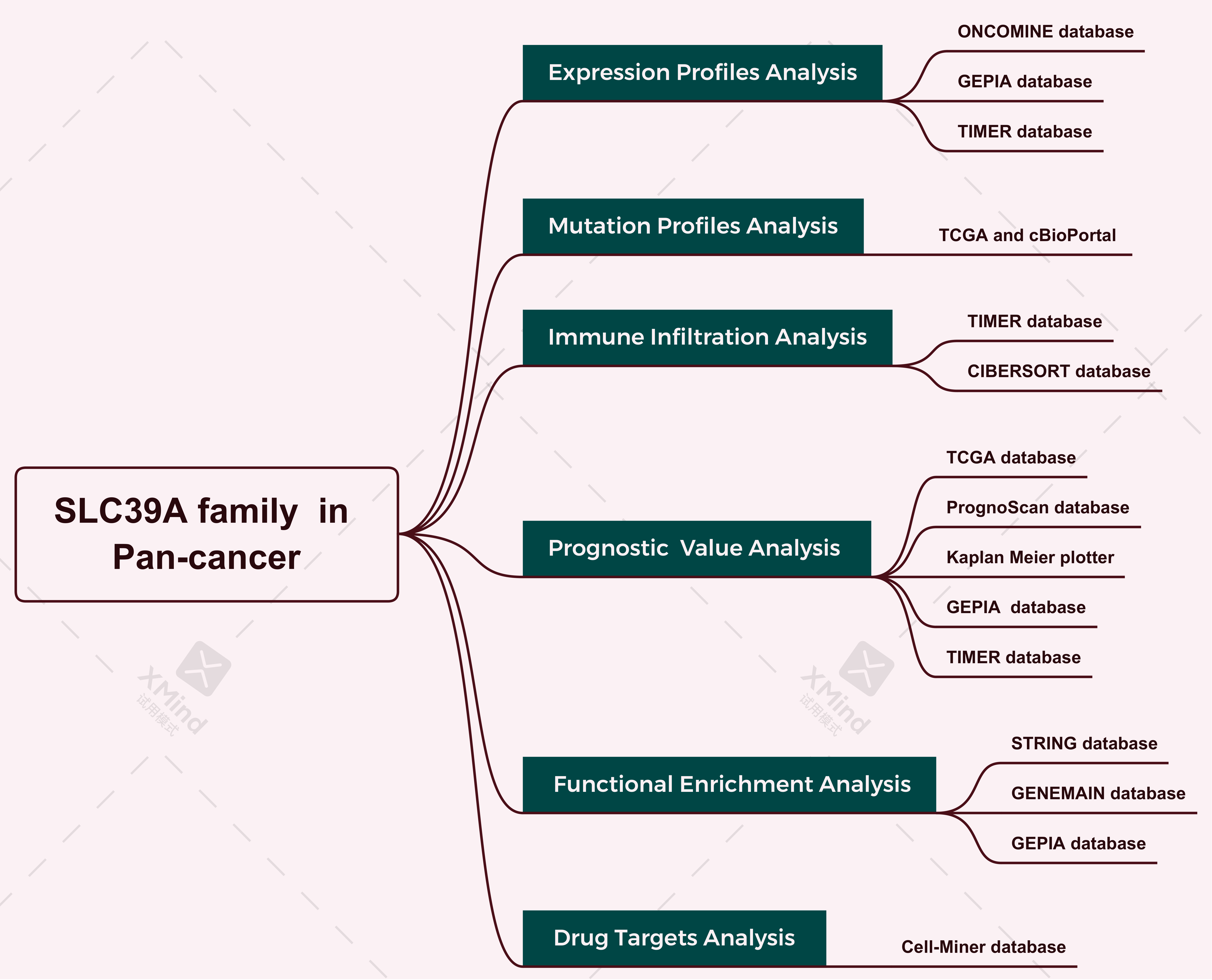


Figure S2. The histogram of the relationship between SLC39A family genes expression and drug sensitivity. The middle line of the box represents the median and the lower and upper bounds represent the 25th and 75th percentiles respectively.*represent the P value = 0.05.


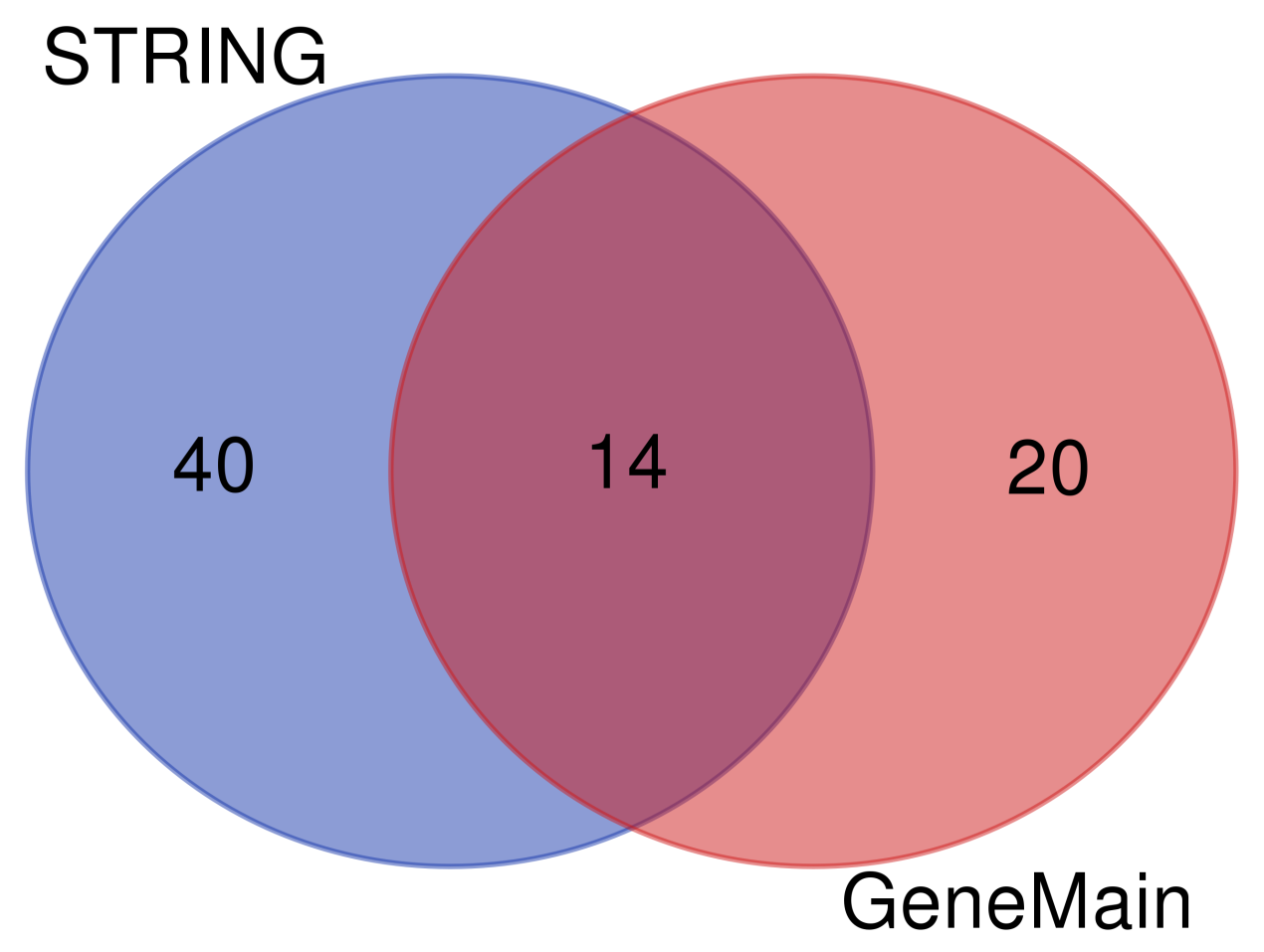


Figure S3. The histogram of the relationship between SLC39A family genes expression and drug sensitivity. The middle line of the box represents the median and the lower and upper bounds represent the 25th and 75th percentiles respectively.*represent the *P* value = 0.05.


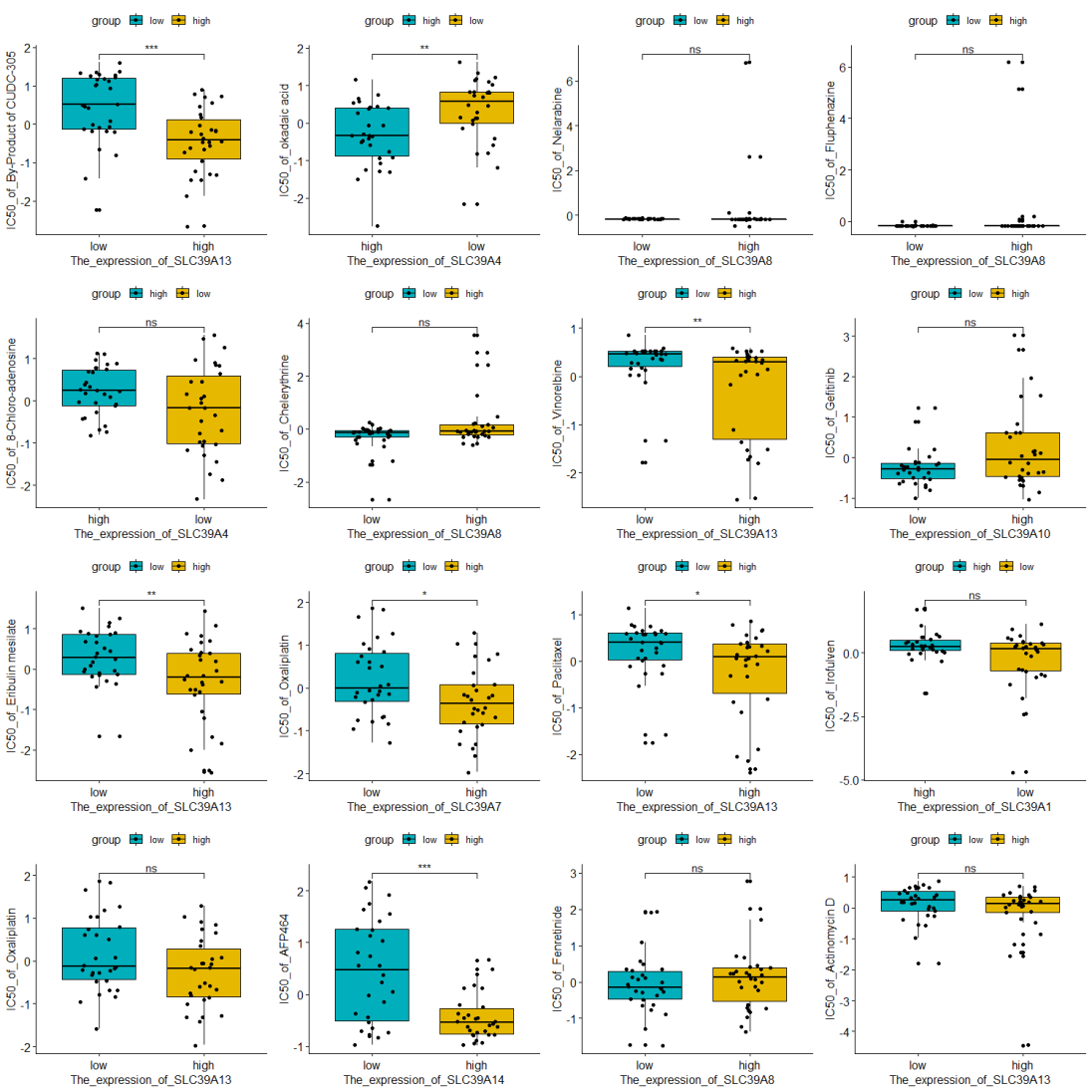

Supplement: Supplementary file 1 [file DataSheet1.docx]
